# Predictors of outcome following neonatal encephalopathy in low- and middle-income countries: a systematic review and meta-analysis

**DOI:** 10.3389/fped.2025.1668799

**Published:** 2025-11-20

**Authors:** Samantha Sadoo, Phillip Wanduru, Eva Loucaides, Carol Nanyunja, Haitao Hu, Emily L. Webb, Hannah Blencowe, Frances M. Cowan, Eric O. Ohuma, Kirsty Le Doare, Cally J. Tann

**Affiliations:** 1 Faculty of Epidemiology and Population Health, London School of Hygiene and Tropical Medicine, London, United Kingdom; 2 NHS Trust Neonatal Department, University College London, London, United Kingdom; 3 Department of Health Policy Planning and Management, Makerere University School of Public Health, Kampala, Uganda; 4 Department of Global Public Health, Karolinska Institutet, Stockholm, Sweden; 5 Institute for Infection and Immunity, City St George’s University, London, United Kingdom; 6 Non-communicable Diseases Department, MRC/UVRI Uganda & LSHTM Research Unit, Entebbe, Uganda; 7 Department of Microbiology, The Chinese University of Hong Kong, Hong Kong, Hong Kong SAR, China; 8 Department of Paediatrics, Imperial College London, London, United Kingdom

**Keywords:** neonatal encephalopathy, low- and middle-income countries, predictors, outcome, death, disability, neurodevelopmental impairment

## Abstract

**Background:**

Intrapartum-related neonatal encephalopathy (NE) is a leading cause of neonatal deaths and childhood-onset developmental disabilities worldwide. Accurate prediction of neurodevelopmental outcomes is crucial to support effective neonatal follow-up strategies, guide parental counselling, and inform future neuroprotection research. Whilst NE disproportionately affects those in low- and middle-income countries (LMICs), existing prognostic accuracy research is primarily based in high-income countries. This systematic review and meta-analysis aims to provide a comprehensive synthesis of the predictors of adverse early childhood outcome following NE in LMICs.

**Methods:**

Four databases were searched, using terms related to “neonate”, “encephalopathy”, “predictor”, “outcome”, and “LMIC”. NE was defined as ≥35 weeks' gestation, evidence of intrapartum event, and abnormal neurology on early clinical assessment. Adverse childhood outcome was defined as neurodevelopmental impairment (assessed using a standardised tool) +/- death, at ≥12 months of age. At least two reviewers performed screening of abstracts and full texts, data extraction, and bias assessment (Quality in Prognosis Studies tool). We reported sensitivity and specificity for each predictive tool, stratifying results by therapeutic hypothermia (TH) status. Meta-analyses were performed where possible. The protocol was registered on PROSPERO in January 2024 (CRD42024485734).

**Results:**

Of the 7,464 articles screened, 32 were included, totalling 1,538 infants with NE from 14 LMICs. Predictors were categorised into neonatal clinical scores for NE severity (16 studies), neurophysiology (13), neuroimaging (14), biomarkers (10), and post-neonatal neurological clinical assessments (5). Highest prognostic accuracy was demonstrated by MRI (moderate to severe abnormalities; sensitivity 69% and specificity 90%), electroencephalography (early severe background abnormality; sensitivity 87% and specificity 93%), Prechtl's General Movements Assessment (absent fidgety movements; sensitivity 78% and specificity 95%), and Hammersmith Infant Neurological Examination (score <67; sensitivity 88–100% and specificity 88%–100%).

**Conclusions:**

A range of standardised tools showed good prognostic accuracy for adverse early childhood outcome following NE. However, this review highlights the paucity of NE research in LMICs using adequate sample sizes and duration of follow-up. Data synthesis and comparability were limited by substantial heterogeneity between study populations, definitions and timing of predictors and outcomes, and variable study quality. Data to evaluate the role of TH on prognostic accuracy were insufficient. Further research to evaluate combinations of the most promising predictors is warranted.

## Introduction

Intrapartum-related neonatal encephalopathy (NE), or newborn brain injury as a result of complications around the time of birth, affects more than one million newborns every year, and is associated with a high risk of neurodevelopmental disabilities such as cerebral palsy (CP), learning difficulties, visual and hearing impairments, and seizure disorders (1). NE is the second leading neurological cause of disability-adjusted life years (DALYs) of any age group worldwide, with the vast majority occurring in low- and middle-income countries (LMICs) where more than a quarter of NE survivors are affected by childhood-onset developmental disabilities (1, 2).

NE is a heterogeneous clinical condition of diverse aetiology that affects the term newborn at or soon after birth, and is characterised by a disturbance of neurological dysfunction, manifesting as reduced level of consciousness, tone and reflexes, difficulties in respiration, and seizures (3). Diagnosis of intrapartum-related NE, or hypoxic–ischaemic encephalopathy (HIE), traditionally require evidence of both neurological dysfunction *and* evidence of an intrapartum hypoxic–ischaemic (HI) event such as placental abruption, foetal or early neonatal acidosis, low Apgar score, or prolonged need for resuscitation after birth (4). Therapeutic hypothermia (TH) is the standard of care in high-income country (HIC) settings, evidenced to improve disability-free survival amongst infants with moderate to severe HIE (5). However, safety and effectiveness in diverse LMIC care contexts is less clear particularly in those without access to neonatal intensive care, and consequently, TH is not consistently applied (6, 7). Several emerging neuroprotective strategies are under active investigation in pre-clinical and clinical studies, but a gap remains for a novel intervention that is feasible and effective in diverse settings (8). Neurodevelopmental follow-up after hospital discharge is crucial to inform the early identification and referral of NE survivors with evolving neurodevelopmental impairment (NDI) to specialised services. In HICs, early intervention strategies for children with NDI have been shown to optimise functional outcomes, taking advantage of the window of early neuroplasticity in those early weeks and months (9). However, in LMICs, resource constraints frequently prevent the conduct of comprehensive surveillance of all at-risk newborns, which risks missing crucial opportunities for early care and support of affected children and their families. Therefore, accurate, early predictors of childhood outcome could facilitate targeted follow-up of those at highest risk, in addition to guiding future clinical trials of neuroprotective strategies through their use as early surrogate measures of long-term outcome.

**Figure 1:**
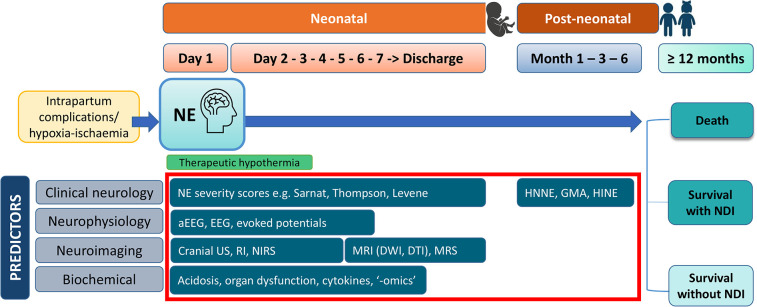
Conceptual framework for the predictors of adverse early childhood outcome following intrapartum-related neonatal encephalopathy. This figure displays predictors that will be specifically evaluated for in this review, within the red box. aEEG, amplitude-integrated encephalography; DTI, diffusion-tensor imaging; DWI, diffusion-weighted imaging; EEG, electroencephalography; GMA, Prechtl's general movements assessment; HINE, Hammersmith Infant Neurological Examination; HNNE, Hammersmith Neonatal Neurological Examination; MRI, magnetic resonance imaging; MRS, magnetic resonance spectroscopy; NDI, neurodevelopmental impairment; NE, intrapartum-related neonatal encephalopathy; NIRS, near-infrared spectroscopy; RI, resistive index; US, ultrasound.

Several predictors of neurodevelopmental outcomes following NE have been identified in HIC cohorts, which include clinical, neurophysiological, neuroimaging, and laboratory markers that are measured during the first days and weeks after birth (Figure 1). Prognostic accuracy has been previously demonstrated for early neonatal clinical assessments of NE severity [Sarnat (10), Thompson (11), and Levene (12)], amplitude-integrated electroencephalography (aEEG) or EEG (13), cranial ultrasound (cUS) (14), magnetic resonance imaging (MRI) (15), and magnetic resonance spectroscopy (MRS) (16), laboratory biomarkers (17), as well as neonatal and infant neurological assessments such as the Hammersmith Neonatal Neurological Examination (HNNE) (18, 19), the Hammersmith Infant Neurological Examination (HINE) (20), and Prechtl's General Movements Assessment (GMA) (21). Predictive performance may differ in LMICs compared with HICs because of differences in the NE population, specifically, the aetiology, nature, and timing of brain injury in diverse socioeconomic and care contexts. TH has been suggested to impact the accuracy of certain predictors, although evidence is limited in this regard (22). Understanding the available evidence on predictors from LMICs has been limited by the use of restrictive criteria in previous systematic reviews that may have hindered the inclusion of studies from LMICs, such as focusing only on therapeutic hypothermia cohorts or predictor modalities not routinely available in diverse settings (23–26).

The aim of this systematic review and meta-analysis was to examine the predictors of adverse outcome following NE in LMICs. Specific objectives were to summarise the available evidence on the prognostic accuracy of neonatal and post-neonatal predictive tools in detecting early childhood neurodevelopmental impairment +/- death amongst NE infants in diverse LMIC contexts, and to identify gaps in the existing evidence base to inform future research.

## Methods

This systematic review and meta-analysis was reported in accordance with the Preferred Reporting Items for Systematic Reviews and Meta-Analyses (PRISMA) 2020 guidelines (Supplementary material S1) (27). The study was registered with PROSPERO on 12th January 2024 (CRD42024485734).

### Data sources and search strategy

A systematic search was conducted on four online databases: Ovid MEDLINE, Ovid Embase, Cochrane Library, and Global Index Medicus, last updated on 12th March 2025. Search strategies were developed in collaboration with experienced university librarians at the London School of Hygiene & Tropical Medicine (LSHTM). Search terms related to “neonatal encephalopathy”, “predictor”, “outcome”, and “low- and middle- income country” were used, including medical subject headings (MESH) terms where possible, and the Cochrane Effective Practice and Organisation of Care (EPOC) LMIC search filter (Supplementary Material S2). The syntax for search terms was adapted for each database. In addition, citations and reference lists of included papers and relevant systematic reviews were manually searched (28). Non-peer-reviewed publications identified through these methods were eligible for screening and potential inclusion.

### Eligibility criteria

The criteria for study inclusion are displayed in Table 1, and the range of predictors specifically evaluated in this review is presented in Figure 1. We focused the scope of this review on validated tools applicable in the neonatal and post-neonatal periods up to 6 months of age, based on evidence from HICs (29–31). These included early neonatal clinical assessments of NE severity (Sarnat staging, Thompson score, Levene score), neurophysiology [aEEG, EEG, evoked potentials (EP), near-infrared spectroscopy (NIRS)], neuroimaging (cUS, MRI, MRS), early biomarkers (circulating markers in blood, urine or cerebrospinal fluid, including acid-base balance, “-omics”), and neonatal/post-neonatal neurological assessments (HNNE, HINE, GMA), as well as prediction models comprising at least two of these tools (Figure 1).

**Table 1:** Eligibility criteria for study inclusion.

Category	Inclusion criteria
Population	≥35 weeks' gestation and/or birthweight ≥1.8 kg (equivalent to term/ near-term infants) Neonatal encephalopathy (meeting both the following A and B criteria^a^): (A) Intrapartum event, defined as any of the following: sentinel event (uterine rupture, antepartum haemorrhage, cord prolapse, placental abruption); obstructed labour; foetal distress (foetal bradycardia, abnormal CTG); need for prolonged resuscitation at birth; Apgar score <7 at ≥5 min; foetal/neonatal acidosis within first hour (pH < 7.1/BE > 12) (B) Abnormal neurological clinical examination including abnormal tone, consciousness, reflexes, seizures, or standardised neurological assessment e.g., Sarnat, Thompson
Setting	Any low- or middle-income country, as defined by the World Bank during the median year of the study (43)
Study type	Original data from randomised or non-randomised controlled trial, cohort, cross-sectional, or case–control studies Conference proceedings, letters, and other non-peer-reviewed publications (where sufficient details were provided/obtained) Year of publication from 2000 onwards
Outcome	Combined death and NDI, or NDI (median age of assessment ≥12 months; NDI defined using a clinically validated instrument)
Predictor	Any clinical assessment, investigative/imaging modality or laboratory test (biomarker) administered between birth and 6 months of age^b^ Sensitivity and specificity for outcome reported^c^

BE, base excess; CTG, cardiotocograph; NDI, neurodevelopmental impairment; NE, intrapartum-related neonatal encephalopathy.

a (A) and (B) are not clear within the study's eligibility criteria, the participant characteristics in the results were reviewed to clarify whether the population fit the above criteria for NE.

b Definition of a predictor should be specified (e.g., units of measurement, timing).

c Alternatively, data provided to construct a 2 × 2 contingency table to enable the calculation of sensitivity and specificity.

The exclusion criteria were as follows: NE cohorts combined with other populations (e.g., preterms) and data not presented separately; infants recruited from subspecialised populations (e.g., mothers with gestational diabetes); and any study with a sample size of less than 15 (number with complete data for predictors and outcomes). For studies reporting data overlapping with another included study, the study with the smaller complete dataset was excluded as a duplicate. No language restrictions were applied at any stage of the review process.

### Literature search and screening

Articles identified from the database searches were exported to EndNote and duplicates removed. Subsequently, articles were imported to Rayyan for further de-duplication and to assist with screening of articles (32). Amongst four reviewers, titles and abstracts were screened independently by at least two reviewers, to determine which should progress to full text review. An initial calibration exercise was conducted on the titles and abstracts of the first 100 articles to check for consensus; the percentage agreement between all four reviewers was 80.0%, after which discrepancies were discussed. Subsequently, one author (SS) reviewed all abstracts, and an independent second review was divided between three reviewers (PW, EL, and CN). Where disagreements occurred, the article progressed to the full text review stage. One author (SS) reviewed all full texts, and the second review was divided equally amongst two reviewers (PW and EL). Foreign language articles were reviewed by authors fluent in the language (French and Spanish by EL, Chinese by HH); for other language articles, Google Translate was utilised. Where full texts were not accessible, corresponding authors were contacted via ResearchGate; if no response was received within 4 weeks, the article was excluded. For excluded full texts, one exclusion reason was recorded per article, ranked in order of animal study, year, LMIC, NE definition, outcome definition, predictor type or definition, or prediction data.

### Data extraction

All data were extracted by one author (SS) onto a pre-designed and piloted Excel spreadsheet (v.2108, Microsoft). In addition, independent data extraction was performed by a second reviewer (PW/EL) for one-third of articles and discrepancies discussed. Subsequently, data extracted from the remaining articles were verified (i.e., not independently extracted) by PW/ EL, because of time constraints, following guidance (33). Prognostic accuracy data were transcribed directly from articles where available, otherwise data were extracted to construct a contingency (2 × 2) table for direct calculation. Data with concerns about accuracy, that is, inconsistencies in reporting, were excluded.

### Risk of bias assessment

Risk of bias (RoB) was assessed for each included study using the Quality in Prognosis Studies (QUIPS) tool, which consists of five domains each with three to seven items: (a) patient selection, (b) study attrition, (c) measurement of prognostic factors, (d) outcome measurements, and (e) statistical analysis and reporting (Supplementary material S3) (34). We excluded the “confounding” domain from the original tool based on confounding being a causal concept and adjustment in predictive studies potentially leading to bias by overfitting (24). Scores were assigned to each item, domain, and finally to the article overall by two reviewers independently (SS, EW/PL), and disagreements discussed.

### Statistical analysis

Narrative synthesis was performed in accordance with guidelines published by Popay et al., utilising summary tables (35). Where sensitivity and specificity were reported within articles, these data were used; where only data on proportions were provided to construct a contingency (2 × 2) table for predictor and outcome variables, the sensitivity and specificity were calculated. Forest plots were used to display sensitivity and specificity with 95% confidence intervals (CI) of predictors from individual studies; the pooled effect size was omitted for studies not deemed to be sufficiently comparable (based on differences in predictor definition/timing). Results were stratified according to the TH status of cohorts (“TH”, “no TH”, or “mixed TH” where only part of the cohort underwent TH). Studies implementing neuroprotective interventions other than whole-body TH were categorised as “no TH.” For interpretation of sensitivity and specificity, excellent/high was defined as 90%–100%, good/very good 80%–90%, fair/moderate 70%–80%, and poor/low <70%. Studies rated as high risk of bias were indicated in the footnotes of forest plots. Definitions of ≥12 month “adverse outcome” for individual studies (NDI, or, combined death/NDI) were listed within forest plot figures; however, stratification of predictors by different types of outcomes was not performed because of heterogeneity. Publication bias could not be assessed using formal tests of asymmetry because of the insufficient number of included studies (36).

Meta-analyses were performed using Stata (version 17) for determining the prevalence of adverse outcomes (NDI or combined death/NDI) and for the predictors of outcome, where data for prognostic accuracy were available for three or more studies with comparable definition and timing of the predictor. Forest plots displayed pooled effect sizes and weights. A random-effects model was used, expecting significant heterogeneity between studies. We utilised the Freeman–Tukey double arcsine transformation as a variance-stabilising measure for meta-analyses of non-normally distributed proportions (37). Meta-analyses were not performed for subgroups according to TH status because of the limited number of studies. Where a study reported prediction for more than one outcome, that is, combined death/NDI as well as NDI in survivors, the larger sample size was included in the meta-analysis (i.e., combined death/NDI).

### Patient and public involvement

The design or reporting of this study did not involve patients or the members of the general public.

### Ethics statement

Ethics approval was not obtained as this study included only those data that were already published.

## Results

**Figure 2:**
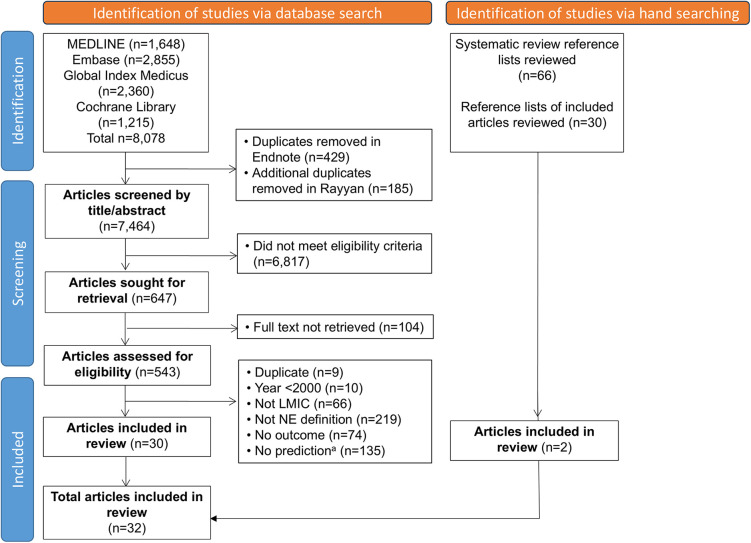
PRISMA flowchart for study selection. ^a^Of these excluded studies, 36 studies provided data for the predictor of neonatal death only; 7 studies provided data for association between an eligible predictor and outcome, e.g., means/medians, *p*-values, odds/risk ratios (but not sensitivity/specificity or 2 × 2 table data); 9 studies provided sensitivity/specificity for outcome, but the predictor was not eligible e.g., maternal/neonatal demographic or risk factor. LMIC, low- and middle-income countries; NE, intrapartum-related neonatal encephalopathy.

Databases were searched on 13th March 2024, and updated on 12th March 2025, yielding a total of 7,464 results after de-duplication (Figure 2). Of the 543 full texts reviewed, 32 were included. Overall, 95.7% agreement was achieved for all screened abstracts and 96.3% agreement at full text screening.

**Figure 3:**
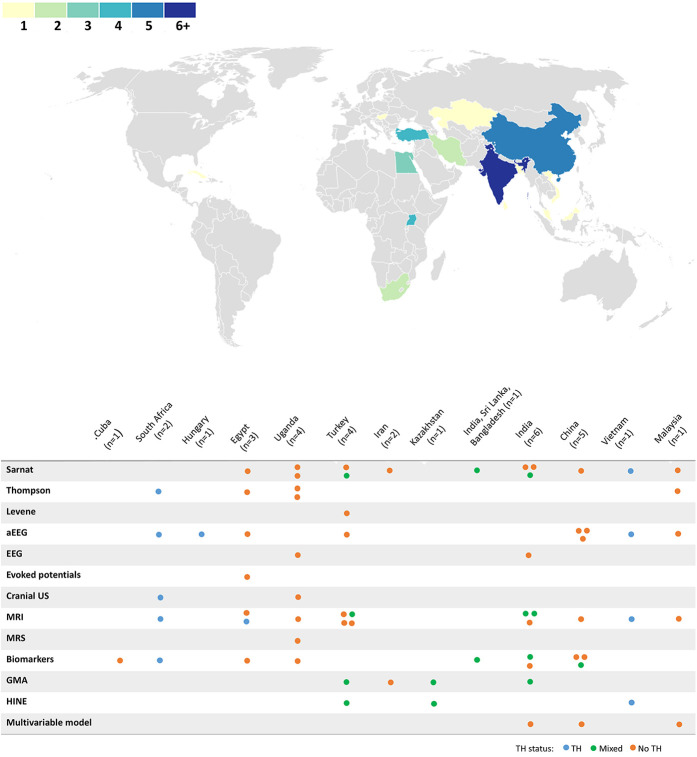
Geographical distribution of included articles, and predictor modalities according to country and therapeutic hypothermia status. The choropleth map displays the number of articles by country, and the table displays the number of articles by country reporting each modality of predictor (one dot is equivalent to one article, coloured according to therapeutic hypothermia status). aEEG, amplitude-integrated encephalography; EEG, electroencephalography; GMA, Prechtl's general movements assessment; HINE, Hammersmith Infant Neurological Examination; MRI, magnetic resonance imaging; MRS, magnetic resonance spectroscopy; NE, intrapartum-related neonatal encephalopathy; TH, therapeutic hypothermia; US, ultrasound.

A total of 1,538 infants with NE were included across 32 articles (30 distinct studies; four articles were based on data from two Ugandan studies (38–41). Study characteristics are given in Table 2 and Supplementary Material S4. Years of recruitment were between 2000 and 2021, and years of publication were between 2004 and 2024. Studies spanning 14 LMICs were included [one was multi-country (42)] (Figures 3A,B). By region [as defined by the World Bank (43)], 14 studies were from Southeast Asia, 6 from sub-Saharan Africa, and the only country classified as low income was Uganda (4 articles, based on 2 studies). Four articles were not written in the English language [three Chinese (44–46) and one Spanish (47)]. One article was a conference abstract (41); the remaining were peer-reviewed publications. Study design was retrospective in 6 studies and prospective in 26 studies [of these, 4 were randomised controlled trials (42, 48–50)]. For the definition of NE, evidence of an intrapartum event was indicated by the Apgar score in the majority of studies (93%), and clinical neurological status was most commonly reported using the modified Sarnat staging (44%), followed by the Thompson score (22%). With regard to neuroprotection status, 12 studies reported the implementation of whole-body TH; six studies in the entire cohort (total 362 infants) and six studies in approximately half of the cohort, of which one study also reported the implementation of selective head cooling (51). One study reported the administration of Erythropoietin in half of the cohort (49). The median number of participants per study was 38.5 (IQR 25–67, range 15–197 participants).

**Table 2:** Characteristics of included studies.

Reference	Study design	Country (language)^a^	Recruitment years	Neuroprotection	Total N^b^	Age of follow-up, outcome assessed (% adverse)	Predictor(s) assessed
Aker et al. (48)	RCT	India	2013–2015	TH vs. none	43	18 m: death/NDI (30%)	MRI, GMA
Apaydın et al. (51)	RC	Turkey	2014–2018	TH vs. head cooling	47	24 m: NDI (19%)	Sarnat, MRI, GMA, HINE
Belet et al. (148)	PC	Turkey	–	None	24	3.5–4 years: NDI (63%)	Sarnat, MRI
Boskabadi et al. (149)	PC	Iran	2013–2017	None	32	24 m: NDI (78%)	Sarnat
Cseko et al. (66)	RC	Hungary	2005–2009	TH	70	18–24 m: death/NDI (37%)	aEEG
El Ayouty et al. (56)	PC	Egypt	2002–2004	None	25	18 m: NDI (72%)	Sarnat, aEEG, MRI
El Beheiry et al. (75)	PC	Egypt	2015–2016	TH	33	12 m: NDI (48%)	MRI, DTI
Gane et al. (50)	RCT	India	2011–2013	TH vs. none	103	12 m: death/NDI (34%)	Biomarker: genomics
Huang et al. (67)	P	China	2013–2020	None	50	18 m: NDI (36%)	Sarnat, aEEG, MRI, biomarkers: pH, NSE, multivariable models
Jia et al. (46)	PC	China [Chinese]	2012–2013	None	83	12 m: NDI (22%)	aEEG
Jiang et al. (45)	PC	China [Chinese]	2010–2013	TH	31	15–18 m: NDI (19%)	Biomarker: GFAP
Jose et al. (58)	PC	India	2010–2011	None	30	12 m: NDI (50%)	EEG, CT, MRI, multivariable models
Kalay et al. (74)	RC	Turkey	2006–2010	None	21	1–4.5 years: NDI (48%)	MRI, DWI
Kali et al. (69)	RC	South Africa	2008–2011	TH	67	12 m: death/NDI (39%)	Thompson, aEEG, RI, MRI
Khedr et al. (68)	PCC	Egypt	–	None	20	12 m: death/NDI (30%)	Thompson, evoked potentials, biomarker: pH
Lally et al. (52)	PC	India	2009	TH vs. none	38	3.5 years: NDI (42%)	Sarnat, MRI
Liu et al. (44)	PC	China [Chinese]	2003–2005	None	25	18 m: death/NDI (24%)	aEEG
Liu and Feng (77)	P	China	2005–2008	None	44	12 m: NDI (32%)	Biomarker: IL-1b
Malla et al. (49)	RCT	India	2012–2015	Epo vs. none	100	18–22 m: death/NDI (55%)	Sarnat
Mathieson et al. (40)	PC	Uganda	2019–2020	None	39	12–24 m: death/NDI (54%)	Thompson, EEG
Mfingwana et al. (76)	RC	South Africa	2008–2011	TH	60	12 m: death/NDI (45%)	Biomarker: nucleated RBCs
Montaldo et al. (42)	RCT	India, Sri lanka, Bangladesh	2015–2019	TH vs. none	45	18–22 m: death/NDI (51%)	Sarnat, biomarker: coagulation
Nanyunja et al. (41)	PC (Conference)	Uganda	2019–2020	None	21	12–24 m: death/NDI (29%)	MRI, MRS
Ong et al. (55)	PC	Malaysia	2000–2001	None	38	12 m: death/NDI (34%)	Sarnat, Thompson, aEEG, cranial US, multivariable models
Pang et al. (39)	PCC	Uganda	2011–2012	None	150	27–30 m: death/NDI (59%)	Biomarker: IL-10
Polat et al. (57)	RC	Turkey	2006–2008	None	25	44–48 m: NDI (24%)	Levene, aEEG, MRI
Preeti et al. (53)	PC	India	2007–2008	None	76	12 m: death/NDI (37%)	Sarnat, biomarkers: pH, base deficit
Soleimani et al. (79)	PCC	Iran	2012–2013	None	15	12–18 m: NDI (67%)	GMA
Tann et al. (38)	PCC	Uganda	2011–2012	None	197	27–30 m: death/NDI (58%)	Sarnat, Thompson, cranial US
Tran et al. (54)	P	Vietnam	2016–2019	TH	101	18 m: death/NDI (64%)	Sarnat, aEEG, MRI, HINE
Velázquez et al. (47)	PC	Cuba (Spanish)	2011–2012	None	25	12 m: NDI (48%)	Biomarker: pH
Zhussupova et al. (78)	PC	Kazakhstan	2020–2021	TH vs. none	31	24 m: CP (29%)	GMA, HINE

aEEG, amplitude-integrated electroencephalogram; CP, cerebral palsy, DTI, diffuse tensor imaging; DWI, diffusion-weighted imaging; EEG, electroencephalography; Epo, erythropoietin; GFAP, glial fibrillary protein; GMA, Prechtl's general movements assessment; HINE, Hammersmith Infants Neurological Examination; IL, interleukin; m, month, MRI, magnetic resonance imaging; MRS, magnetic resonance spectroscopy; NDI, neurodevelopmental impairment; NE, intrapartum-related neonatal encephalopathy; nRBCo, nucleated red blood cells; NSE, neuron-specific enolase; PC, prospective cohort; PCC, prospective case–control; RC, retrospective cohort; RCT, randomised controlled trial; TH, therapeutic hypothermia; US, ultrasound.

a Language stated if not in English.

b Total number of participants in the study who had complete data for prediction of outcome (highest number from the list of predictors in the last column).

The age of follow-up for NDI assessment ranged between 1 and 4.5 years of age. Four studies reported both outcomes of combined death/NDI and NDI in survivors (38, 52–54). Standardised neurodevelopment tools used to evaluate NDI in the included studies were as follows: Bayley Scales of Infant and Toddler Development 2nd or 3rd edition (BSID-II *n* = 5, BSID-III *n* = 8), Gross Motor Function Classification System (GMFCS, *n* = 6), Denver Developmental Screening Test (DDST-II, *n* = 5), Amiel-Tison Neurological Assessment (*n* = 3), HINE (*n* = 3), Developmental Assessment Scale for Indian Infants (DAS-II, *n* = 2), Griffiths Mental Developmental Scales 2nd edition (GMDS-II, *n* = 1), Ages and Stages Questionnaire (ASQ, *n* = 1), Infant Neurological International Battery Test (Infanib, *n* = 1), Ankara Developmental Screening Inventory (ADSI, *n* = 1), Children's Development Center of China Infants Intelligence Development Test (CDCC IDT, *n* = 1), Gesell Developmental Schedules (*n* = 1), and Bax criteria (*n* = 1). In addition, some definitions of NDI included epilepsy, occipito-frontal head circumference, and vision and hearing loss.

### Risk of bias assessment

Fourteen studies were rated as low RoB, 8 moderate, and 10 high (Supplementary Material S6). Amongst studies rated high RoB, the most frequently reported domains with moderate or high RoB were statistical analysis and presentation (*n* = 9), and study attrition (*n* = 7). The agreement score for overall RoB rating was 78% between two reviewers.

### Prevalence of adverse outcomes

Acknowledging high heterogeneity (i^2^ > 80%), the pooled prevalence rate of composite death/NDI outcome (14 studies) was 43% (95% CI 36%–50%). The prevalence rate of NDI in survivors (16 studies) was also 43% (95% CI 33%–52%) (Supplementary Material S5).

### Neonatal clinical scoring systems for determining the severity of NE

#### Modified Sarnat staging

**Figure 4:**
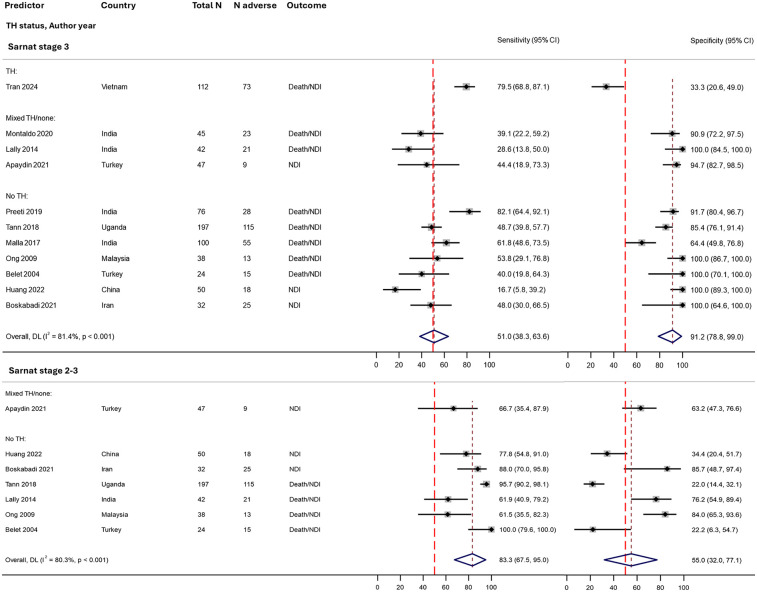
Forest plots assessing the performance of the modified Sarnat staging in predicting early childhood outcome following NE, stratified by therapeutic hypothermia status, with meta-analysis. Further details on study characteristics and definitions of predictors and outcomes are presented in Supplementary Material S4. Studies rated as overall high risk of bias were Belet et al. (148) and Boskabadi et al. (149) (Supplementary Material S6). TH, therapeutic hypothermia administered to the entire cohort; mixed TH/none, therapeutic hypothermia administered to approximately half of the cohort (and no TH in the remaining); no TH, no therapeutic hypothermia administered; 95% CI, 95% confidence interval; N adverse, number of infants with death/NDI or NDI; NDI, neurodevelopmental impairment; NE, intrapartum-related neonatal encephalopathy.

Overall, 11 studies provided data to assess a Sarnat stage 3 (peak stage during admission) as a predictor of NDI or death/NDI outcomes (total 763 infants from 7 countries; Figure 3, Table 2); one study reported sensitivity and specificity, whilst the remaining provided 2 × 2 table data for calculation (Supplementary Material S4) (55). Heterogeneity between studies was high (i^2^ > 80%). Pooled sensitivity was poor (51%, 95% CI 38%–64%); however, specificity was good (91%, 95% CI 71%–99%) (Figure 4). Only two studies demonstrated good sensitivity, whilst nine studies showed excellent specificity. The one TH study showed a particularly low specificity for death/NDI (54).

Conversely, amongst the six studies (430 infants) assessing Sarnat stage 2–3 (vs. Sarnat stage 1), pooled sensitivity was very good (83%, 95% CI 68%–95%), but at the expense of specificity (55%, 95% CI 32%–77%) (Figure 4).

#### Thompson score

**Figure 5:**
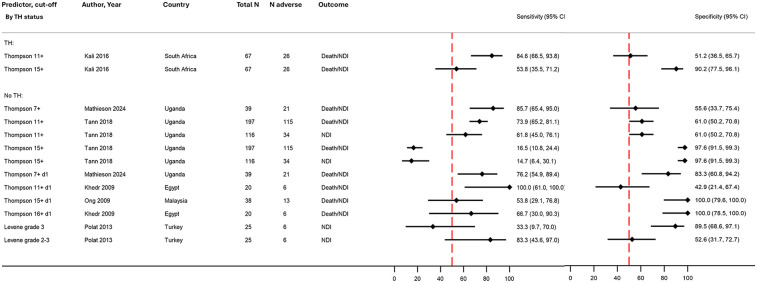
Forest plot assessing the performance of the Thompson and Levene scores in predicting adverse early childhood outcome following NE, stratified by therapeutic hypothermia status (no meta-analysis). Further details on study characteristics and definitions of predictors and outcomes are presented in Supplementary Material S4. No studies were rated as overall high risk of bias*.* TH, therapeutic hypothermia administered to the entire cohort; mixed TH/none, therapeutic hypothermia administered to approximately half of the cohort (and no TH in the remaining); no TH, no therapeutic hypothermia administered; 95% CI, 95% confidence interval; N adverse, number of infants with death/NDI or NDI; NDI, neurodevelopmental impairment; NE, intrapartum-related neonatal encephalopathy.

In total, five studies provided data to assess the prognostic accuracy of the Thompson score for adverse outcomes (361 infants, 4 countries; Figure 3, Table 2). Of these, two studies reported sensitivity and specificity, whilst the rest reported 2 × 2 table data (Supplementary Material S4) (40, 55). For calculating the peak score during hospital admission, three cut-offs were used (7+, 11+, and 15+); with increasing thresholds, the sensitivity reduced but specificity increased (from excellent to poor and vice versa) (Figure 5). For day 1 scores, the prognostic accuracy was more variable. One study assessing both death/NDI and NDI found similar prognostic accuracy for both outcomes (Figure 5) (38).

#### Levene score

Only one study utilised the Levene score, finding a similar trend as the modified Sarnat staging for prediction of outcomes; grade 3 showed poor sensitivity and high specificity, whilst the reverse was seen for grade 2 and 3 (Figure 5) (57).

### Neurophysiology

#### aEEG and EEG

Twelve studies reported the predictive value of amplitude-integrated EEG or EEG (a/EEG) for adverse outcomes (564 infants from 9 LMICs; Figure 3). Two used EEG and the remaining 10 used aEEG (Table 2) (40, 58). All but two studies referenced specific criteria for reporting the background pattern; three used the classification of Hellström-Westas and Rosén (59), three studies referenced Sefton (60), and one study each referenced a scoring system adapted from Murray et al. (61), Al Naqeeb et al. (62), Laroia et al. (63), Biagioni et al. (64), and Liu et al. (65) (Supplementary Material S4). With regard to the timing of a/EEG, nine studies reported on early background abnormalities within 12 h of birth, and nine studies reported on abnormalities over the first week after birth.

**Figure 6:**
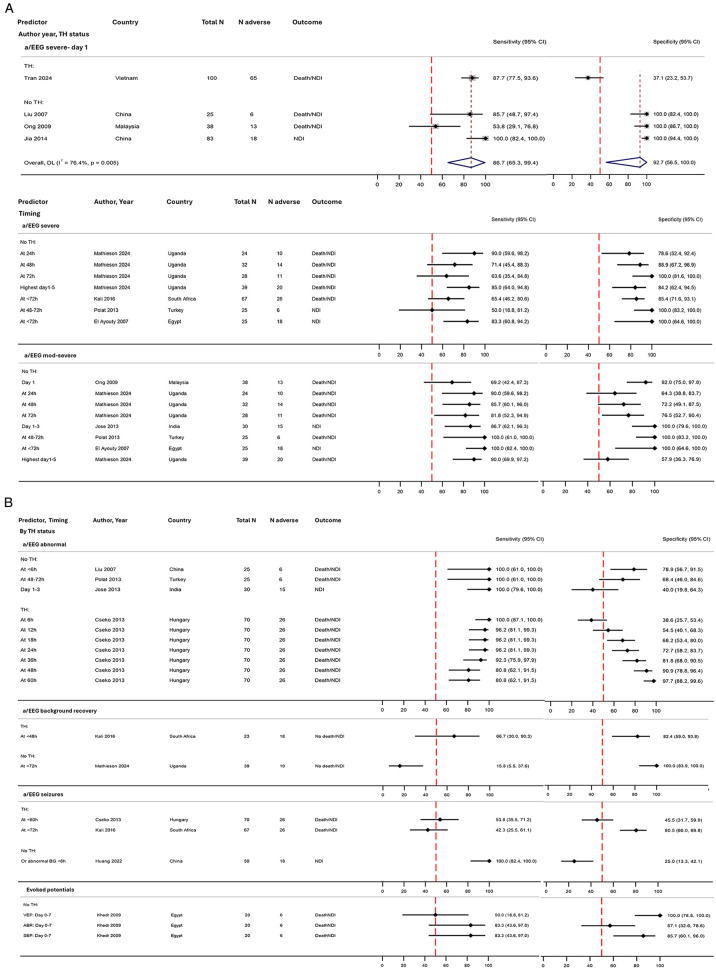
Forest plots assessing the performance of neurophysiological tools in predicting adverse early childhood outcome following NE, stratified by therapeutic hypothermia status. **(A)** a/EEG moderate to severe background abnormalities with and without meta-analyses and **(B)** a/EEG abnormalities (no meta-analysis). Further details on study characteristics and definitions of predictors and outcomes are presented in Supplementary Material S4. Studies rated as overall high risk of bias were Jia et al. (46) and Liu et al. (44) (Supplementary Material S6). TH, therapeutic hypothermia administered to the entire cohort; mixed TH/none, therapeutic hypothermia administered to approximately half of the cohort (and no TH in the remaining); no TH, no therapeutic hypothermia administered; ABR, auditory brainstem responses; aEEG, amplitude-integrated electroencephalography; BG, background; 95% CI, 95% confidence interval; N adverse, number of infants with death/NDI or NDI; NDI, neurodevelopmental impairment; NE, intrapartum-related neonatal encephalopathy; SEP, somatosensory evoked potentials; VEP, visual evoked potentials.

Four studies reported severe background abnormalities on day 1 (246 neonates, from three countries in Southeast/East Asia). Pooled prognostic accuracy was excellent (sensitivity 87%, 95% CI 65%–99%; specificity 93%, 95% CI 57%–100%) (Figure 6A). The one TH study showed a notably low specificity (54). Amongst the additional four studies assessing severe background activity over the first few days, sensitivity was variable (50%–90%), but specificity was overall good (79%–100%) (Figure 6A). Moderate to severe background abnormality showed good sensitivity in four out of five studies (82%–100%) aside from the study reporting early (day 1) changes (55), and specificity was also excellent for 4 out of 5 studies (92%–100%). Multiple timepoints across the first 5 days were assessed by two studies [one TH (66) and one no TH (40)]; both demonstrated a clear pattern of decreasing sensitivity and increasing specificity over time. Recovery of EEG background abnormality was reported by two studies, defined as normalisation from an initial severe background to predict a favourable outcome (no death/NDI); whilst specificity was good, sensitivity was poor (Figure 6B). Only three studies assessed electrographic seizures, one of which combined seizures with background abnormality (67); prognostic accuracy varied but overall poor (Figure 6B).

#### Evoked potentials

EPs were reported by one small study without TH; of the three modalities, somatosensory demonstrated the highest overall prognostic accuracy (sensitivity 83% and specificity 86%) (Figure 6B) (68).

### Neuroimaging

Fifteen studies reported data assessing the prognostic accuracy of neuroimaging for adverse outcomes (Table 2, Figure 3, Supplementary Material S4).

#### Cranial ultrasound

**Figure 7:**
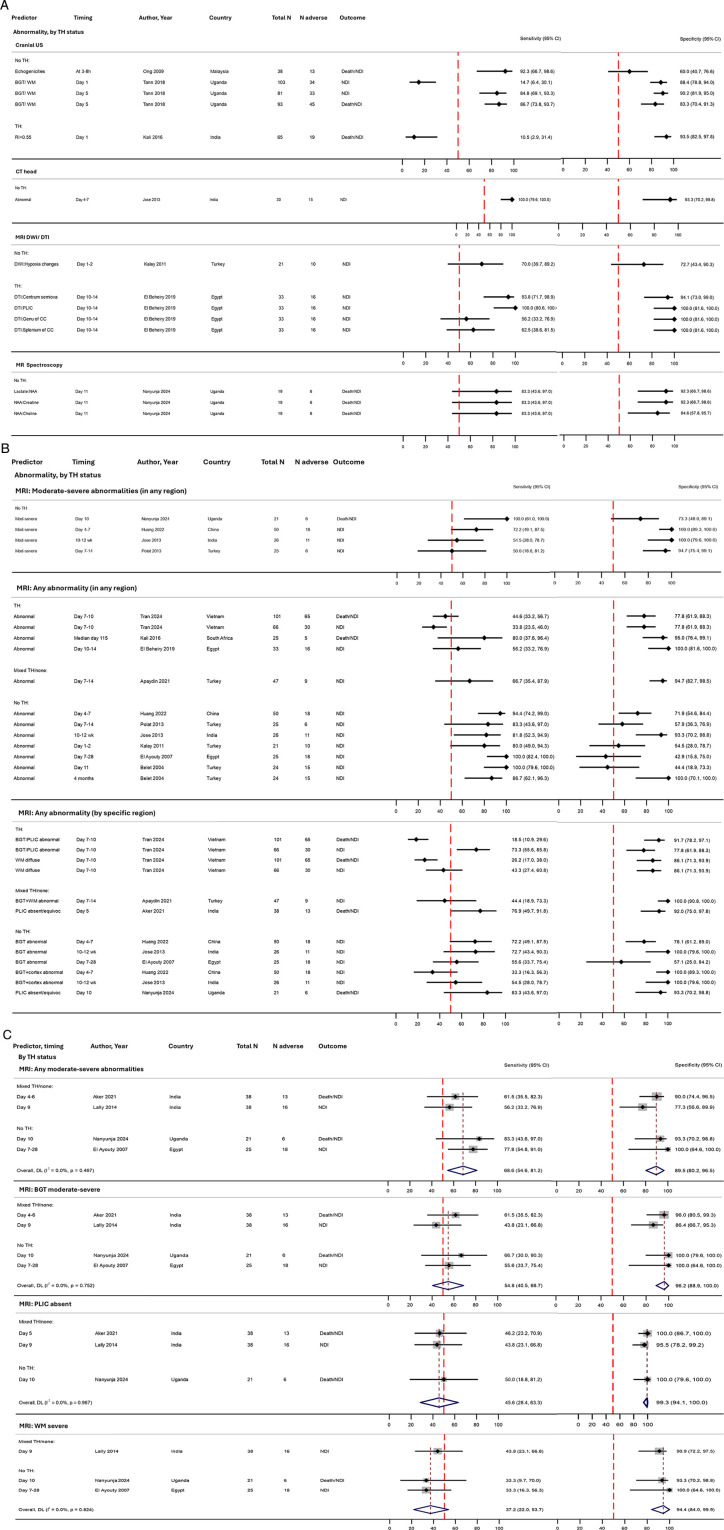
Forest plots assessing the performance of neuroimaging in predicting adverse early childhood outcome following NE, stratified by therapeutic hypothermia status: **(A)** cranial ultrasound, CT, MRI, and MRS (no meta-analysis), **(B)** MRI abnormalities (no meta-analysis), and **(C)** MRI moderate to severe abnormalities, with meta-analysis. Further details on study characteristics and definitions of predictors and outcomes are presented in Supplementary Material S4. Tran et al. (54) assessed death/NDI and NDI outcomes; both are presented in this figure. Studies rated as overall high risk of bias are Belet et al. (148), El Beheiry et al. (75), Jose et al. (58), Kalay et al. (74), and Liu et al. (44) (Supplementary Material S6). **(B)** “Mod-severe” was defined as moderate to severe BGT or absent PLIC or severe WM [Nanyunja et al. (41), Aker et al. (48), Lally et al. (52)], or moderate to severe BGT or severe WM [El Ayouty et al. (56)]. **(C)** “Mod-severe” was defined as: NICHD grade 2A/2B/3 [Nanyunja et al. (41)], Barkovich grade 3–4 [Huang et al. (67), Jose et al. (58)], and severe changes in brainstem, ventral cerebellar vermis, BGT, or perirolandic regions [Polat et al. (57)]. “Any abnormality” was defined as abnormal BGT/PLIC or diffuse WM injury [Tran et al. (54)]; BGT or cortex [Huang et al. (67), Jose et al. (58)]; BGT, PLIC, WM, cortex, brainstem [El Beheiry et al. (75)]; BGT, PLIC, WM, cortex [Kali et al. (69)]; BGT, cortex, brainstem, parasagittal [Polat et al. (57)]; “hypoxia changes” [Kalay et al. (74)]; and grey matter, WM, atrophy, encephalomalacia [Belet et al. (148)]. TH, therapeutic hypothermia administered to the entire cohort; mixed TH/none, therapeutic hypothermia administered to approximately half of the cohort (and no TH in the remaining); no TH, no therapeutic hypothermia administered; BGT, basal ganglia and thalami; CC, corpus callosum; 95% CI, 95% confidence interval; CT, computed tomography; cUS, cranial ultrasound; DTI, diffusion-tensor imaging; DWI, diffusion-weighted imaging; mod, moderate; MRI, magnetic resonance imaging; MRS, magnetic resonance spectroscopy; N adverse, number of infants with death/NDI or NDI; NAA, N-acetyl aspartate; NDI, neurodevelopmental impairment; NE, intrapartum-related neonatal encephalopathy; PLIC, posterior limb of the internal capsule; SH, selective head cooling; T1/T2-W, T1 and T2-weighted imaging; WM, white matter.

Three studies presented cUS findings; of these, one reported only resistive indices (RIs). Two studies assessed day 1 abnormalities; Ong et al. found that any echogenicities or ventricular dilatation had excellent sensitivity (92%) but poor specificity (60%) for death/NDI, whilst Tann et al. found that bilateral basal ganglia and thalami (BGT) and/or diffuse white matter (WM) changes predicted NDI with very poor sensitivity (15%) but good specificity (88%) (Figure 7A) (38, 55). On day 4–5, Tann et al. found that sensitivity increased substantially (85%) and specificity was maintained (90%) (38). Kali et al. found that RI on day 1 had very poor sensitivity but high specificity (94%) for death/NDI (Figure 7A) (69).

#### CT head

Jose et al. performed a CT head in the first week, reporting an excellent prognostic accuracy of cerebral oedema, hypodensities, and/or bleeds for NDI (Figure 7A) (58).

#### Magnetic resonance imaging

Conventional T1- and T2-weighted MRI findings were reported by 14 studies. The majority of studies performed MRI within the first 2 weeks after birth, most commonly around day 10; three studies performed scans at later timepoints [El Ayouty et al. (56) at 7–28 days; Jose et al. (58) at 10–12 weeks; Kali et al. (69) at median 115 days, range 4–150] (Figure 7B). A variety of scoring criteria were used, most commonly the Rutherford criteria (70), by four studies (Supplementary Material S4), although there were still differences in reporting; Aker et al. (48), Lally et al. (52), and Nanyunja et al. (41) defined moderate to severe changes according to the original Rutherford (70) criteria [moderate to severe BGT, absent posterior limb of the internal capsule (PLIC), and/or severe WM], whilst Kali et al. (69) reported only “any” abnormality. Nanyunja et al. (41) also assessed the total injury score, which was a later addition to the Rutherford criteria by Thoresen et al. (71). El Ayouty et al. (56) did not reference specific scoring criteria but provided a comparable description of moderate to severe changes (moderate to severe BGT and severe WM, although there is no mention of PLIC) (Supplementary Material S4).

Amongst four studies (122 infants, majority without TH), moderate to severe changes in the BGT, PLIC, and/or WM demonstrated only fair pooled sensitivity (69%, 95% CI 55%–81%) but high specificity (90%, 95% CI 80%–97%) (Figure 7C). Assessing the predictive value of these regions individually, it was found that pooled sensitivities were poor for all regions (between 37% and 55%; highest for the BGT), but pooled specificities were consistently excellent (94%–99%; highest for the PLIC) (Figure 7C). Two studies [one TH (48) and one no TH (41)] found that combining absent with equivocal signal in the PLIC improved sensitivity to 77%–83%, whilst maintaining excellent sensitivity (Figure 7B). The Barkovich criteria (72) was used by two studies; however, the timing of scans was much later in Jose et al. (58) than in Huang et al. (67) (Supplementary material S4). In both studies, an abnormal BGT had reasonable sensitivity (72%–73%) with improved specificity (78%–100%); when the BGT was combined with cortex changes, specificity was excellent but sensitivity poor (33%–55%) (Figure 7B). Only one study (41) utilised the NICHD criteria (73), finding grade 2A/2B/3 to be perfectly sensitive but only moderately specific (73%) (Figure 7B). Tran et al. assessed both death/NDI and NDI outcomes; whilst there were some differences, no clear trend was seen (54).

#### Diffusion-weighted imaging

One small study presented results specifically from diffusion-weighted imaging (DWI); they found that “diffusion limitation consistent with hypoxia” on early day 1–2 scan had only fair predictive value (70% sensitivity and 73% specificity) for NDI (Figure 7A) (74).

#### Diffusion-tensor imaging

One study reported on diffusion-tensor imaging (DTI), finding excellent prognostic accuracy of PLIC and centrum semiovale abnormalities for NDI, whilst the corpus callosum had poor sensitivity (Figure 7A) (75).

#### Magnetic resonance spectroscopy

One small study (41) found high prognostic accuracy for all three evaluated peak-area metabolite ratios: lactate/N-acetyl aspartate (NAA), NAA/creatine, and NAA/choline (sensitivities 83%, specificities 85%–92%) (Figure 7A).

### Biomarkers

Eleven distinct biomarkers were reported by 10 studies for prediction of adverse outcomes from 8 LMICs (Table 2, Figure 3). All biomarkers were tested from blood samples collected within the first few days after birth from the umbilical cord or the neonate (Supplementary Material S4**)**.

#### Markers of acid-base balance

**Figure 8:**
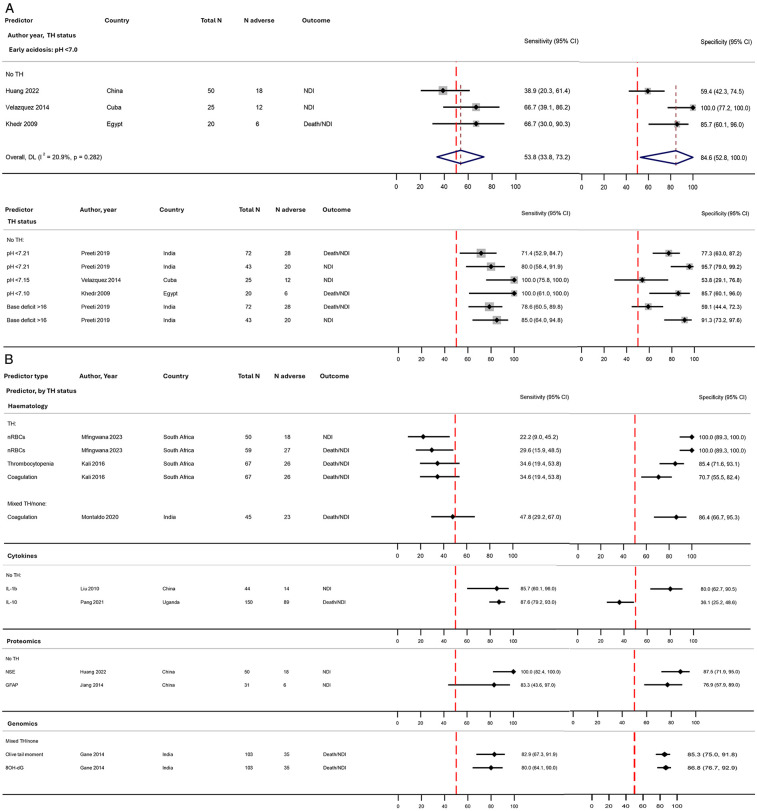
Forest plots assessing the performance of biomarkers in predicting early childhood adverse outcome following NE, stratified by therapeutic hypothermia status: **(A)** early acidosis with and without meta-analysis and **(B)** other biomarkers (no meta-analysis). Further details on study characteristics and definitions of predictors and outcomes are presented in Supplementary Material S4. Preeti et al. (53) assessed prognostic accuracy for both death/NDI and NDI outcomes in the same cohort (both are shown in the figure). Studies rated as overall high risk of bias were Liu and Feng (77) and Mfingwana et al. (76) (Supplementary Material S6). TH, therapeutic hypothermia administered to the entire cohort; mixed TH/none, therapeutic hypothermia administered to approximately half of the cohort (and no TH in the remaining); no TH, no therapeutic hypothermia administered; 8-OHdG, 8-hydroxy2-deoxyguanosine; 95% CI, 95% confidence interval; GFAP, glial fibrillary acidic protein; IL, interleukin; mod, moderate; N adverse, number of infants with death/NDI or NDI; NDI, neurodevelopmental impairment; NE, intrapartum-related neonatal encephalopathy; NSE, neuron-specific enolase.

Three studies (95 neonates) reported early (first hour) severe acidosis with pH <7.0. Pooled sensitivity was poor (54%, 95% CI 34%–73%) but specificity was good (85%, 95% CI 53%–100%) (Figure 8A). At higher pH thresholds (<7.1–7.2), sensitivity improved (71%–100%) and specificity was maintained (Figure 8A). One study assessed base deficit to predict both death/NDI and NDI outcomes; for NDI, prognostic accuracy was excellent (sensitivity 85% and specificity 91%) (Figure 8B) (53).

#### Haematological markers

Three haematological biomarkers were reported in four studies: early nucleated red blood cells (76), thrombocytopenia (69), and coagulation abnormality (42, 69); all had poor sensitivity (22%–48%) but fair to excellent specificity (71%–100%) (Figure 8B).

#### Cytokines

Two cytokines were reported by one study each; IL-1b (cord blood) had very good sensitivity and specificity (89% and 81%, respectively) (77), whilst IL-10 (within 12 h of birth) had very good sensitivity but poor specificity (89% and 36%, respectively) (39) (Figure 8B).

#### Proteomics

Serum levels of two brain-specific proteins were reported by one study each; neuron-specific enolase (NSE, day 3) had excellent sensitivity and specificity (100% and 88%, respectively) (67), and glial fibrillary acidic protein (GFAP, 6–12 h) showed good sensitivity and specificity (77% and 78%) (45) (Figure 8B).

#### Genomics

One “Indian” study reported two markers of DNA damage (within the first 36 h) and found very good prognostic accuracy (sensitivity 80%–85% and specificity 85%–88%) (Figure 8B) (50).

### Post-neonatal clinical assessments

#### Prechtl’s general movements assessment

**Figure 9:**
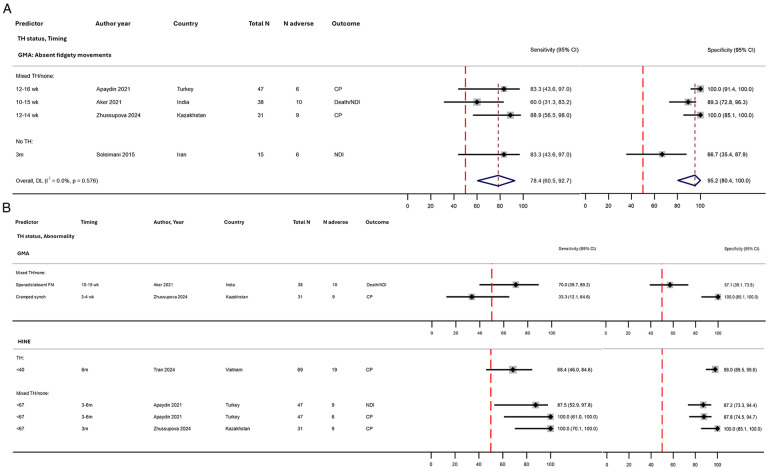
Forest plots assessing the performance of post-neonatal clinical neurological assessments in predicting early childhood adverse outcome following NE, stratified by therapeutic hypothermia status: **(A)** absent fidgety movements on GMA with meta-analysis and **(B)** GMA and HINE (no meta-analysis). Further details on study characteristics and definitions of predictors and outcomes are presented in Supplementary Material S4. Apaydın et al. (51) assessed CP and NDI outcomes; both are shown in this figure. No studies were rated as overall high risk of bias*.* TH, therapeutic hypothermia administered to the entire cohort; mixed TH/none, therapeutic hypothermia administered to approximately half of the cohort (and no TH in the remaining); no TH, no therapeutic hypothermia administered; 95% CI, 95% confidence interval; CP, cerebral palsy; FM, fidgety movements; GMA, general movements assessment; HINE, Hammersmith Infant Neurological Examination; mod, moderate; m, months; N adverse, number of infants with death/NDI or NDI; NE, intrapartum-related neonatal encephalopathy; NDI, neurodevelopmental impairment; synch, synchronised; wk, weeks.

Four small studies assessed the predictive performance of the GMA for a 12–24-month outcome, totalling 131 infants from India, Iran, Kazakhstan, and Turkey (Table 2, Figure 3, Supplementary Material S4**)**. Absent fidgety movements at 10–16 weeks showed good pooled sensitivity (78%, 95% CI 61%–93%) and excellent specificity (95%, 95% CI 80%–100%) for adverse outcomes (Figure 9A). For the two studies evaluating the prediction of CP specifically (51, 78), predictive performance appeared higher overall compared with death/NDI (48) or NDI (79) outcomes. Combining absent with sporadic fidgety movements reduced the specificity (Figure 9B) (48). One study reported that cramped synchronised movements at 3–4 weeks had perfect specificity but very poor sensitivity (33%) (Figure 9B) (78).

#### Hammersmith infant neurological examination

Three studies reported on the prognostic accuracy of the HINE, one each at 3 months (78), 6 months (54), and 3–6 months of age (51) (Figure 9B). A score of <67 was strongly sensitive and specific (87%–100%) for cerebral palsy/NDI in two studies, whilst a much lower cut-off of <40 showed poor sensitivity (54). One study (51) reporting the prediction of cerebral palsy with NDI more broadly found a higher sensitivity for CP (100% vs. 88%); nevertheless, predictive performance was very good for both.

### Multivariable prediction models

Three studies reported sensitivity and specificity for a combination of predictors (Table 2) (55, 58, 67). A total of eight different models were presented, most commonly featuring EEG (seven models), followed by the Thompson score, MRI, and cranial US (three models each) (Supplementary Material S4). The most predictive combination consisted of aEEG (abnormal, at <6 h), MRI (abnormal, day 4–7), and serum NSE (≥27.3 μg/L, day 3) (Table 3) (67).

**Table 3:** Assessing the performance of prediction models for adverse early childhood outcome following NE.

Reference	Predictors in model	Outcome	Total	Events	Sensitivity	Specificity
			*N*	*N*	(95% CI)	(95% CI)
Huang et al. (67)	EEG, MRI	NDI	50	18	94.5 (74–99)	79.98 (61–89)
	EEG, MRI, NSE	NDI	50	18	100 (82–100)	97.7 (84–99)
Jose et al. (58)	EEG, CT	NDI	31	15	100 (80–100)	33.3 (14–56)
	EEG, MRI	NDI	26	11	100 (74–100)	40 (20–64)
Ong et al. (55)	Thompson, EEG	Death/NDI	38	13	100 (77–100)	80.6 (61–91)
	Thompson, cranial US	Death/NDI	38	13	100 (77–100)	80.6 (61–91)
	EEG, cranial US	Death/NDI	38	13	100 (77–100)	80.6 (61–91)
	Thompson, EEG, cranial US	Death/NDI	38	13	100 (77–100)	80.6 (61–91)

95% CI, 95% confidence interval; CT, computed tomography head; EEG, electroencephalography; Events, number of infants with adverse events (death/NDI or NDI); MRI, magnetic resonance imaging; NDI, neurodevelopmental impairment; NSE, serum neuron-specific enolase; US, ultrasound.

Sensitivity and specificity values are in percentage. 95% confidence intervals for all models were manually calculated based on available data as they were not reported within the articles. Studies rated as overall high risk of bias were Jose et al. (58) (Supplementary Material S6).

## Discussion

To our knowledge, this is the first systematic review and meta-analysis assessing a range of predictive tools for detecting adverse neurodevelopmental outcomes following NE specifically in LMICs. Accurate, early prediction of outcomes is a priority for clinicians, researchers, and families. In the preceding paragraphs, we focused on the predictors of long-term disability +/- death outcomes determined at ≥12 months of age, with the primary aim of informing surveillance and triage of NE infants with neurodevelopmental concerns to specialised services and early childhood intervention programmes. Our review highlighted several tools with promising prognostic accuracy, but further research is needed to confirm these findings in large cohorts in diverse contexts. Individual modalities demonstrated to have the highest overall prognostic accuracy for adverse outcomes by more than one study were severe background abnormality on EEG (day 1), moderate to severe abnormalities on MRI (neonatal period), absent fidgety movements on GMA (12–16 weeks), and suboptimal HINE score (3–6 months). Promising tools with very good predictive performance but reported only in a single study each were somatosensory evoked potentials (42), cranial US (38), MRS (41), biomarkers IL-1b (77), NSE (67), and GFAP (45), and genomics-related tools (50). In addition, multivariable predictive models comprising combinations of clinical assessments, EEG, neuroimaging, and biomarkers were demonstrated by three studies to have excellent prognostic accuracy (55, 58, 67). Considering clinical feasibility in addition to the statistical strength of prediction will be crucial in informing the application of any early identification strategy in diverse LMIC contexts.

Differences in the prognostic value of tools between LMIC and HIC settings may be in part explained by data sparsity, variable quality, or feasibility of implementation in LMICs. The different timing of predictors is a key factor that must be taken into consideration. These differences may also be attributed to differences in the population resulting from diverse socioeconomic risk factors and differences in NE definition due to a lack of blood gas availability and challenges in accurate determination of gestational age where access to antenatal care is limited. Neuroprotection status may also contribute to differences, although insufficient data limit evaluation. We compare our findings from LMIC settings with the wider literature from HICs.

### Prevalence of adverse outcomes

The pooled prevalence of death/NDI at 18 months in the major TH trials was 31.7% in TH infants and 61.4% in non-TH infants, which is comparable to our review of LMIC studies (43% overall) (Supplementary Material S5) (5). For NDI in survivors, only one of the TH trials (CoolCap) contributed data finding a prevalence of 13% in TH and 27.8% in non-TH infants; in comparison, we found a higher NDI prevalence (43% overall) (80). Whilst we stratified prevalence according to TH status (Supplementary Material S5), insufficient data prevent conclusions from being drawn on the effectiveness of TH in LMICs (81). Notably, despite our review including only those infants who had complete data on the predictors of outcome, the rate of prevalence of death/NDI was similar to that reported in a recent systematic review on NE outcomes in LMICs (44.6%) (82).

### Clinical assessments of NE severity

Whilst Sarnat staging was primarily developed for determining the severity of NE, higher severity has been associated with worse outcomes in HICs (5, 10). A total Sarnat score has also been evaluated, summating individual category scores to achieve a total of 0–18; the MARBLE study found that on admission, sensitivity was poor (39%) but specificity was good (84%) (83). The Thompson score has also demonstrated good predictive performance for long-term outcomes, with a higher score resulting in a trade-off in sensitivity (reducing) and specificity (increasing), similar to that seen in our study (11, 84, 85). The Levene score is less commonly used and therefore data are limited (86); first described in 1985 as a simplified version of Sarnat (four items), there is a need for a validated simplified clinical score for use in resource-constrained settings (12).

Timing of assessment probably influences the prognostic value of early clinical assessments (84, 87). The NICHD and CoolCap TH trials found that the modified Sarnat was more predictive at later timepoints than on admission (88, 89); in our review, day 1 Sarnat [only one study (56)] was less predictive, but no studies provided data on serial assessments.

### Neurophysiology

The predictive performances of EEG and aEEG have been evaluated by several systematic reviews and meta-analyses, consisting predominantly of studies from HICs (25, 26, 90–93). Our findings align with the wider literature, that severe background abnormalities (burst suppression or flat trace) are strongly predictive of adverse outcomes. TH is recognised to reduce the predictive performance of early EEG (93); the only TH cohort in our meta-analysis had a notably low specificity (37%) (54). The systematic reviews by Ouwehand et al. (26) and Chandrasekaran et al. (93) systematic reviews found that sensitivity was higher at earlier timepoints; conversely, specificity increased at later timepoints, and that the optimal balance was achieved around 36 h (26), as we also found in the two studies that evaluated multiple timepoints (40, 66). However, a meta-analysis by Han et al. found the reverse (day 1 severe background was more specific, whereas beyond 24 h was more sensitive) (90). A variety of EEG scoring systems were used in our studies, but we were not able to assess differences in prediction; one study found similar predictive value irrespective of whether it used the Hellström-Westas or Al Naqeeb criteria (94). Not only is severity of background an important feature but its evolution as well; however, this aspect is less well reported. We found only two small studies assessing normalisation of a severe background over the first days, although other HIC studies have found good predictive accuracy of background recovery and time-to-sleep-wake cycle (94, 95). Whilst EEG is accepted as the gold standard for monitoring brain activity, Ouwehand et al. (26) reported no difference in the prognostic accuracy of background abnormalities on EEG compared with aEEG, although another meta-analysis by Liu et al. (25) found EEG to be more specific, whilst aEEG was more sensitive (25); we were not able to evaluate this because only two studies reported the use of EEG. Whilst seizure burden is a recognised predictor of outcome, our review identified three studies assessing only the presence of electrographic seizures, which showed overall poor prognostic accuracy; this has also been shown in HIC studies (29, 92).

Evoked potentials are used infrequently, even though practically they can be added to standard EEG setups; our review included only one small study, finding good predictive performance of somatosensory EPs. Liu et al.'s systematic review identified five studies (all HICs with TH), finding that overall SEPs had poor prognostic accuracy, although acknowledging that the studies were small (25).

### Neuroimaging

Cranial ultrasound imaging is widely used in diverse settings for early assessment in NE because of its accessibility enabling early and serial imaging at the cotside, yet studies evaluating predictive performance for long-term outcomes are lacking (31). Ouwehand et al.'s systematic review (only TH cohorts) did not identify any eligible studies (26); our review identified only two studies. Only the later scan (day 5) was predictive, probably due to US changes evolving over 24–48 h after the insult (14). The evidence for measurement of RI using Doppler US is stronger; a 2022 systematic review found very good sensitivity (83%) and specificity (92%) across 10 uncooled cohorts for death/severe NDI, although predictive value may be reduced in TH (96). CT was assessed by one study in our review (58), but this modality is not recommended for neonates because of poor parenchymal contrast resolution of the neonatal brain, combined with higher radiation exposure (97).

MRI is the gold standard investigation for NE assessment and prognostication, and excellent prognostic accuracy has been demonstrated by several systematic reviews and meta-analyses, in both TH and non-TH infants (25, 26, 92). Prognostic value varies according to the pattern of injury; central grey nuclei injury (the BGT and PLIC) are well evidenced in HICs to be strongly associated with motor impairment, whilst it is suggested that watershed injury (cortex and adjacent subcortical white matter) is more likely to be associated with cognitive impairment (97); we were not able to evaluate the prediction of different types of outcomes because of the lack of available data. A range of scoring systems are available for MRI reporting; studies in our review utilised the Rutherford et al. (70), Barkovich et al. (72), NICHD (73), and Thoresen et al. (71) scores, but several others have been evaluated, such as the Trivedi et al. (98) and Weeke et al. (99) scores. A systematic review by Langeslag et al. (24) reported similar predictive performance between these scores; recent studies have supported higher prognostic accuracy of the Weeke score (100–102). Timing is a key consideration for prognostication with MRI as with other tools; early scans in the first few days are useful for evaluating the timing of injury but may underestimate injury severity whilst it is still evolving; thus, imaging in the second week is recommended for defining the extent of injury (4). However, two studies comparing MRI at early (first week) and later timepoints (beyond first week) concluded that early scans were more predictive (70). We could not evaluate the effect of timing as most studies performed MRI from the second week onwards, potentially because in LMIC settings without intensive care and other resource constraints, there are challenges in transporting acutely unwell neonates to the scanner (41, 56). Several HIC studies have shown that TH does not significantly impact the predictive performance of MRI for NE outcomes (70, 103, 104).

DWI and DTI are less frequently used adjunct sequences to standard MRI protocols but are useful in prognostication as they provide quantitative assessment [with apparent diffusion coefficient (ADC) maps and fractional anisotropy values, respectively]). In the review by Ouwehand et al., of 22 MRI studies, only 5 reported quantitative findings from DWI and 6 from DTI (26). A meta-analysis of DWI studies found that decreased ADC values in the thalami had high specificity with reasonable sensitivity for adverse outcomes (26); other studies have reported good predictive accuracy in the BGT, PLIC, corpus callosum, centrum semiovale, and WM; but not in the cortex, cerebellum, and brainstem (26, 105, 106). In our review, we discovered that only one small study assessed DWI, finding reasonable prognostic accuracy, but specific ADC values were not reported (74). For abnormalities on DTI, good predictive performance has been reported in various regions, including the BGT, PLIC, anterior LIC, corpus callosum, frontal WM, and corticospinal tracts (26, 95, 107). Because of the phenomena of “pseudonormalisation,” predictive features of diffusion imaging change over time; consistent with this, one study found that ADC values were reduced in the BGT and PLIC on days 2–3 but were increased on day 10 in those with adverse outcomes (108).

MRS is strongly evidenced in HICs to predict adverse outcomes, often more accurately than conventional MRI or EEG (109). The most commonly reported peak-area ratios (the signal amplitude of one metabolite compared with another) are lactate/NAA, NAA/creatine, and NAA/choline ratios (16, 110, 111); these were reported in the one conference abstract in our review (41). Other MRS-derived biomarkers found to be predictive include total thalamic NAA, basal ganglia NAA, NAA plus N-acetylaspartylglutamate (NAAG), and glycerophosphorylcholine plus phosphatidylcholine (GPC + PCh) (112). The importance of timing is related to the expected lactate peak during the first week after a hypoxic–ischaemic insult and subsequent decline towards normal levels by weeks 2–3, whilst in contrast NAA, a marker of neuronal integrity, the levels decline over a few days after the hypoxic–ischaemic insult then remain low, and thus, they may be more predictive on later scans (109, 110). One small study in our review (41) found that whilst lactate/NAA was predictive, the cut-off was substantially lower than seen in HIC cohorts (109); this may have been partly explained by the later timing of imaging. NIRS is a bedside neuroimaging technique that monitors cerebral perfusion and oxygenation, for which good prognostic accuracy has been demonstrated for NE outcomes in HICs (113), but our review did not identify any eligible studies from LMICs.

### Biomarkers

Numerous biochemical markers have been studied in infants with NE; however, evidence is mixed and no consensus has been achieved on which are most predictive at which timepoints. The most common biomarkers in NE are cord/early neonatal pH and base deficit (29, 114), also found in our review. Whilst cut-off values used in TH eligibility criteria are often used for prognostication (pH <7.0 or base deficit ≥16), large population studies suggest that at less severely abnormal pH values, the prediction of short-term outcomes improves (115, 116). Nucleated red blood cells may rise early in severe NE because of rapid erythropoiesis as part of the foetal response to HI (117), and association with 2-year outcomes has been demonstrated (118, 119). Thrombocytopenia has been linked to NE severity, playing a key role in inflammatory processes following HI, and coagulopathy has been linked to both severity and early mortality probably because of multi-organ dysfunction including liver dysfunction, consumptive coagulopathy, and impaired enzymatic activity within the coagulation cascade (120–122). We found poor sensitivity (although very good specificity) for these haematological markers in our review, although studies were small and cut-offs often not clearly defined.

Cytokines are a key element of the inflammatory cascade following HI, and many have shown promising predictive value in the literature: IL-1, IL-6, IL-8, IL-10, IL-13, Il-16, TNF-alpha, interferon-γ, vascular endothelial growth factor, Regulated upon Activation Normal T-Cell Expressed and Secretes (RANTES), and monocyte chemotactic protein (MCP-1) (123, 124–127). A few studies have tested combinations of biomarkers; McGowan et al. found that a panel of cytokines was more predictive than any single marker (126). They also evaluated cytokines at different timepoints, acknowledging the rapidly evolving pathophysiological processes following HI and fluctuations in levels of different biomarkers (17). Serum NSE is one of the most commonly studied brain-specific proteins, which has shown promising association with outcomes in both serum and cerebrospinal fluid (CSF), although CSF is impractical to obtain in many settings (128–131). GFAP has been well studied in adult and paediatric traumatic brain injury (17) and has demonstrated association with short-term outcomes, but long-term outcome studies are needed (127, 132). We found good prognostic accuracy for these brain-specific protein markers, although studies were limited. Genomics in NE is still in its infancy, but promising research is underway (133, 134). One study in our review found good prognostic value for measures to control DNA damage (50), which is probably caused by free radicals released after the occurrence of HI.

### Post-neonatal neurological assessments

Whilst Prechtl's GMA has a wealth of evidence to support the prediction of cerebral palsy in at-risk children more broadly (135, 136), there are limited studies specifically involving NE infants. A 2021 scoping review (137) identified only three NE studies, of which one recruited as early as the 1980s (138). Absent fidgety movements during the “fidgety” development stage (12–16 weeks) were found by Glass et al. (139) to have high specificity (98%) but low sensitivity (50%) for cerebral palsy following NE, whilst a more recent study found high sensitivity and specificity (both 89%) (140). Our meta-analysis of four studies found a slightly lower sensitivity (76%), although this was brought down by one study that predicted combined death/NDI rather than cerebral palsy specifically (48). Ferrari et al. (141) found that cramped synchronised movements during the earlier “writhing” period was highly sensitive (100%) but only moderately specific (69%), which was in contrast to the finding of one study in our review (78). As the GMA has considerable potential for routine use in diverse settings because of its advantages of brevity (1–3 min), non-invasiveness, and being conducive to retrospective or remote interpretation using video recordings, further studies in LMICs are warranted (138).

The HINE is also well evidenced in a broader group of at-risk children, rather than in NE specifically (136, 142). Only three LMIC studies were included in our review (51, 54, 78); whilst we restricted our eligibility to assessments ≤6 months to be a useful predictor of early childhood outcomes, and the HINE is validated for use up to 24 months (20), during screening, we did not find any other articles evaluating prediction beyond 6 months. Two HIC studies have reported excellent prognostic accuracy for cerebral palsy following NE: Moss et al. (143) reported sensitivities ranging between 82% and 90% and specificities between 95% and 100% at 3, 6, and 9 months, and Romeo et al. (144) found a positive predictive value (PPV) and negative predictive value (NPV) of 100% at a later age of 12 months. Two studies in our review (78, 51) showed excellent predictive performance, despite using a higher cut-off total score (<67) than recommended for the age group of 3–6 months. A simplified version of the HINE (“Brief-HINE”) has been developed, reducing the number of items from 26 to 11, whilst maintaining excellent predictive performance; this would be particularly useful to evaluate in a resource-limited setting (145). The HNNE (19), a similar tool designed for use from birth to 1–2 months of age, is widely employed, but studies examining its prognostic accuracy for NE outcomes are limited (146); our review did not find any LMIC studies.

### Multivariable prediction models

Combining predictors is well recognised to strengthen predictive performance, yet our review found only three studies that evaluated multivariable models (55, 58, 67) despite 21 studies reporting data for more than one predictor. A recent systematic review by Langeslag et al. (24) highlighted the paucity of studies assessing prediction models for outcomes after NE; of nine included studies between 2009 and 2021, eight were from HIC TH cohorts and one from a middle-income uncooled cohort in Iran (excluded from our study as some of the cohorts were stated not to have HIE) (140). The median number of predictors included in the models was 4 (range 2–6), and neurological examination and imaging tools were most commonly used, which are similar to our findings. The review by Langeslag et al. highlighted key limitations of existing models as mostly single-centre and retrospective, timing not specified for all predictors (only reported by one model), heterogeneous outcomes, and underpowered (24). In HIC settings, the strong predictive value of the combination of MRI, GMA, and HINE has been frequently reported (9, 147).

### Limitations

This study has several important limitations that may affect the generalisability and interpretation of the findings. Data extraction and risk of bias assessment were only partially independent for all studies, although this method is supported by guidance (33). Small sample sizes and wide confidence intervals in many included studies limited precision, and the requirement for complete data may have introduced selection bias. Studies from low-income countries were scarce, with only four articles from two studies (both Ugandan) included; therefore, we were not able to evaluate differences between low-income and middle-income country settings. It was not possible to evaluate the impact of TH on prognostic accuracy because of limited data. Interpretation was also complicated by heterogeneity in definitions of NE, predictors, and neurodevelopmental outcomes, with the use of over ten different neurodevelopmental assessment tools often before the age of 18 months, and the use of a composite adverse outcome (death/NDI) in nearly half of the studies. Finally, potential publication bias and limited access to full texts, particularly from smaller LMIC journals, may have affected comprehensiveness.

## Conclusions

There is an urgent need to improve prediction of outcomes following NE particularly in LMICs, to support targeted follow-up of NE infants and inform future neuroprotection research. This review summarised the available evidence base from diverse LMIC settings, highlighting the most promising predictors (EEG, MRI, GMA, and HINE), although there was insufficient evidence to support the recommendation of a single predictor, nor the effect of TH on prognostic accuracy. We have identified those that warrant further research in LMICs, with the aim of strengthening the evidence for single or combinations of predictors that are feasible, accessible, and validated for use across diverse populations in LMICs.

## Data Availability

The original contributions presented in the study are included in the article/Supplementary Material, and further inquiries can be directed to the corresponding author.
