# Supplementary material for: Predictors of outcome following neonatal encephalopathy in low- and middle-income countries: a systematic review and meta-analysis
Source: Front Pediatr. 2025 Nov 20;13:1668799. doi: 10.3389/fped.2025.1668799 (PMC12690394; doi:10.3389/fped.2025.1668799)
Supplement: Supplementary file 1 [file Supplementaryfile1.docx]

Contents

[Supplementary material 1: Preferred Reporting Items for Systematic reviews and Meta-analyses (PRISMA) checklist 2](#_Toc211332805)

[Supplementary material 2: Search strategies for each database 5](#_Toc211332806)

[a. Ovid MEDLINE 5](#_Toc211332807)

[b. EMBASE 6](#_Toc211332808)

[c. Cochrane Library 7](#_Toc211332809)

[d. Global Index Medicus 9](#_Toc211332810)

[Supplementary material 3: Quality in Prognosis Studies (QUIPS) risk of bias scoring proforma 10](#_Toc211332811)

[Supplementary material 4: Characteristics and predictor data reported in included articles 12](#_Toc211332812)

[Supplementary material 5: Meta-analyses of prevalence of adverse early childhood outcome after NE in included articles: [A] Composite death and neurodevelopmental impairment [B] Neurodevelopmental impairment (in survivors) 26](#_Toc211332813)

[Supplementary material 6: Risk of bias assessment for included articles 28](#_Toc211332814)

Supplementary material 1: Preferred Reporting Items for Systematic reviews and Meta-analyses (PRISMA) checklist

| **Section and Topic** | **Item no.** | **Checklist item** | **Page number** |
| --- | --- | --- | --- |
| **TITLE** | | | |
| **Title** | 1 | Identify the report as a systematic review. |  |
| **ABSTRACT** | | | |
| **Abstract** | 2 | See the PRISMA 2020 for Abstracts checklist. |  |
| **INTRODUCTION** |  |  |  |
| **Rationale** | 3 | Describe the rationale for the review in the context of existing knowledge. |  |
| **Objectives** | 4 | Provide an explicit statement of the objective(s) or question(s) the review addresses. |  |
| **METHODS** | | | |
| **Eligibility criteria** | 5 | Specify the inclusion and exclusion criteria for the review and how studies were grouped for the syntheses. | Table 1 |
| **Information sources** | 6 | Specify all databases, registers, websites, organisations, reference lists and other sources searched or consulted to identify studies. Specify the date when each source was last searched or consulted. |  |
| **Search strategy** | 7 | Present the full search strategies for all databases, registers and websites, including any filters and limits used. | Supplementary material 2 |
| **Selection process** | 8 | Specify the methods used to decide whether a study met the inclusion criteria of the review, including how many reviewers screened each record and each report retrieved, whether they worked independently, and if applicable, details of automation tools used in the process. |  |
| **Data collection process** | 9 | Specify the methods used to collect data from reports, including how many reviewers collected data from each report, whether they worked independently, any processes for obtaining or confirming data from study investigators, and if applicable, details of automation tools used in the process. |  |
| **Data items** | 10a | List and define all outcomes for which data were sought. Specify whether all results that were compatible with each outcome domain in each study were sought (e.g. for all measures, time points, analyses), and if not, the methods used to decide which results to collect. | Table 1 |
|  | 10b | List and define all other variables for which data were sought (e.g. participant and intervention characteristics, funding sources). Describe any assumptions made about any missing or unclear information. | Figure 1 |
| **Study risk of bias assessment** | 11 | Specify the methods used to assess risk of bias in the included studies, including details of the tool(s) used, how many reviewers assessed each study and whether they worked independently, and if applicable, details of automation tools used in the process. | Supplementary material 3 |
| **Effect measures** | 12 | Specify for each outcome the effect measure(s) (e.g. risk ratio, mean difference) used in the synthesis or presentation of results. |  |
| **Synthesis methods** | 13a | Describe the processes used to decide which studies were eligible for each synthesis (e.g. tabulating the study intervention characteristics and comparing against the planned groups for each synthesis (item #5)). |  |
|  | 13b | Describe any methods required to prepare the data for presentation or synthesis, such as handling of missing summary statistics, or data conversions. |  |
|  | 13c | Describe any methods used to tabulate or visually display results of individual studies and syntheses. |  |
|  | 13d | Describe any methods used to synthesize results and provide a rationale for the choice(s). If meta-analysis was performed, describe the model(s), method(s) to identify the presence and extent of statistical heterogeneity, and software package(s) used. |  |
|  | 13e | Describe any methods used to explore possible causes of heterogeneity among study results (e.g. subgroup analysis, meta-regression). |  |
|  | 13f | Describe any sensitivity analyses conducted to assess robustness of the synthesized results. |  |
| **Reporting bias assessment** | 14 | Describe any methods used to assess risk of bias due to missing results in a synthesis (arising from reporting biases). | Supplementary material 3 |
| **Certainty assessment** | 15 | Describe any methods used to assess certainty (or confidence) in the body of evidence for an outcome. | - |
| **RESULTS** | | | |
| **Study selection** | 16a | Describe the results of the search and selection process, from the number of records identified in the search to the number of studies included in the review, ideally using a flow diagram. | Figure 2 |
|  | 16b | Cite studies that might appear to meet the inclusion criteria, but which were excluded, and explain why they were excluded. | Figure 2 |
| **Study characteristics** | 17 | Cite each included study and present its characteristics. | Table 2, Figure 3, Supplementary material 4 |
| **Risk of bias in studies** | 18 | Present assessments of risk of bias for each included study. | Supplementary material 6 |
| **Results of individual studies** | 19 | For all outcomes, present, for each study: (a) summary statistics for each group (where appropriate) and (b) an effect estimate and its precision (e.g. confidence/credible interval), ideally using structured tables or plots. | Table 3,  Figures 4-9, Supplementary material 5 |
| **Results of syntheses** | 20a | For each synthesis, briefly summarise the characteristics and risk of bias among contributing studies. | Table 3,  Figures 4-9 |
|  | 20b | Present results of all statistical syntheses conducted. If meta-analysis was done, present for each the summary estimate and its precision (e.g. confidence/credible interval) and measures of statistical heterogeneity. If comparing groups, describe the direction of the effect. | Table 3,  Figures 4-9, Supplementary material 5 |
|  | 20c | Present results of all investigations of possible causes of heterogeneity among study results. | - |
|  | 20d | Present results of all sensitivity analyses conducted to assess the robustness of the synthesized results. | - |
| **Reporting biases** | 21 | Present assessments of risk of bias due to missing results (arising from reporting biases) for each synthesis assessed. | - |
| **Certainty of evidence** | 22 | Present assessments of certainty (or confidence) in the body of evidence for each outcome assessed. | - |
| **DISCUSSION** | | | |
| **Discussion** | 23a | Provide a general interpretation of the results in the context of other evidence. |  |
|  | 23b | Discuss any limitations of the evidence included in the review. |  |
|  | 23c | Discuss any limitations of the review processes used. |  |
|  | 23d | Discuss implications of the results for practice, policy, and future research. |  |
| **OTHER INFORMATION** | | | |
| **Registration and protocol** | 24a | Provide registration information for the review, including register name and registration number, or state that the review was not registered. |  |
|  | 24b | Indicate where the review protocol can be accessed, or state that a protocol was not prepared. |  |
|  | 24c | Describe and explain any amendments to information provided at registration or in the protocol. |  |
| **Support** | 25 | Describe sources of financial or non-financial support for the review, and the role of the funders or sponsors in the review. |  |
| **Competing interests** | 26 | Declare any competing interests of review authors. |  |
| **Availability of data, code and other materials** | 27 | Report which of the following are publicly available and where they can be found: template data collection forms; data extracted from included studies; data used for all analyses; analytic code; any other materials used in the review. |  |

*Reference: Page MJ, McKenzie JE, Bossuyt PM, Boutron I, Hoffmann TC, Mulrow CD, et al. The PRISMA 2020 statement: an updated guideline for reporting systematic reviews. BMJ 2021;372:n71*

Supplementary material 2: Search strategies for each database

The search strategy was developed in Ovid MEDLINE and adapted for subsequent databases – EMBASE, Cochrane Library, and Global Index Medicus.

### Ovid MEDLINE

| **Construct** | **Search terms** |
| --- | --- |
| Exposure terms  (neonatal encephalopathy) | (Asphyxia Neonatorum/ or Newborn hypoxia/) OR ("Birth asphyxia".mp.) OR (((Neonat* or newborn or infant* or intrapartum or intrapartum-related or perinatal or baby or babies) adj3 (encephalopath* or hypoxi* or asphyxia* or hypoxia-isch?emi# or hypoxic-isch?emi# or HIE or "brain injury" or "therapeutic hypothermia")).mp) |
|  | AND |
| Outcome terms | ((outcome* or death* or mortalit* or morbidit* or surviv* or disabilit* or impair* or development* or neurodevelopment*).mp. ) OR (mortality/ or "cause of death"/ or child mortality/ or fatal outcome/ or hospital mortality/ or infant mortality/ or perinatal mortality/ or survival rate/) OR (death/ or brain death/ or infant death/ or perinatal death/) |
|  | AND |
| Predictor terms | (Predict* or risk* or factor* or prognos* or associat* or relat* or variable* or marker or diagnos* or detect* or identif* or sensitiv* or model* or score* or algorithm*).mp. |
|  | AND |
| Geographical terms (LMICs) | ((afghanistan or albania or algeria or angola or antigua or barbuda or argentina or armenia or armenian or aruba or azerbaijan or bahrain or bangladesh or barbados or belarus or byelarus or belorussia or byelorussian or belize or benin or dahomey or bhutan or bolivia or bosnia or herzegovina or botswana or bechuanaland or brazil or brasil or bulgaria or "burkina faso" or "burkina fasso" or "upper volta" or burundi or urundi or "cabo verde" or "cape verde" or cambodia or kampuchea or "khmer republic" or cameroon or cameron or cameroun or "central african republic" or "ubangi shari" or chad or chile or china or colombia or comoros or "comoro islands" or "iles comores" or mayotte or "drc" or congo or zaire or "costa rica" or "cote d'ivoire" or "coted' ivoire" or "cote divoire" or "cote d ivoire" or "ivory coast" or croatia or cuba or cyprus or "czech republic" or czechoslovakia or djibouti or somaliland or dominica or "dominican republic" or ecuador or egypt or "united arab republic" or "el Salvador" or "equatorial guinea" or guinea or eritrea or estonia or eswatini or swaziland or ethiopia or fiji or gabon or "gabonese republic" or gambia or georgia or ghana or gibraltar or greece or grenada or guam or guatemala or guinea or "guinea Bissau" or guyana or guiana or haiti or hispaniola or honduras or hungary or india or indonesia or timor or iran or iraq or jamaica or jordan or kazakhstan or kazakh or kenya or korea or kosovo or kyrgyzstan or kirghizia or kirgizstan or "kyrgyz republic" or kirghiz or laos or "lao pdr" or "lao people's democratic republic" or latvia or lebanon or lesotho or basutoland or liberia or libya or lithuania or macau or macao or macedonia or madagascaror malagasy republic or malawi or nyasaland or malaysia or "malay federation" or "malaya federation" or maldives or "indian ocean" or mali or malta or micronesia or kiribati or "marshall islands" or nauru or "northern mariana islands" or palau or tuvalu or mauritania or mauritius or mexico or moldova or mongolia or montenegro or morocco or ifni or mozambique or myanmar or burma or namibia or nepal or antilles or nicaragua or niger or nigeria or oman or muscat or pakistan or panama or "papua new guinea" or "new guinea" or paraguay or peru).mp.) OR ((philippines or philipines or phillipines or phillippines or poland or "polish people's republic" or portugal or "portuguese republic" or "puerto rico" or romania or russia or "russian federation" or ussr or "soviet union" or "union of soviet socialist republics" or rwanda or ruanda or samoa or "pacific islands" or polynesia or "samoan islands" or "navigator island" or "navigator islands" or "sao tome and principe" or "saudi arabia" or senegal or serbia or seychelles or "sierra leone" or slovakia or "slovak republic" or slovenia or melanesia or "solomon island" or "solomon islands" or "norfolk island" or "norfolk islands" or somalia or "south Africa" or "south sudan" or "sri lanka" or ceylon or "saint kitts and nevis" or "st.kitts and nevis" or "saint lucia" or "st. lucia" or "saint vincent and the grenadines" or "saint Vincent" or "st. vincent" or grenadines or sudan or suriname or surinam or guiana or syria or "syrian arab republic" or tajikistan or tadjikistan or tadzhikistan or tadzhik or tanzania or tanganyika or thailand or siam or "timor leste" or "east timor" or togo or "togolese republic" or tonga or "trinidad and tobago" or "trinidad or Tobago" or tunisia or turkey or turkmenistan or turkmen or uganda or ukraine or uruguay or uzbekistan or uzbekor vanuatu or venezuela or vietnam or "west bank" or gaza or palestine or yemen or yugoslavia or zambia or zimbabwe or Rhodesia).mp) OR (("sub-saharan" or subsaharan or "sub saharan" or Africa or sahara or "west indies" or caribbean or "south america" or "south asia" or "middle east" or "developing country" or "third world" or "under developed country" or "underdeveloped country" or "middle income country" or "middle income nation" or "low income country" or "low income nation" or "low income setting" or "low income context" or "middle income setting" or "middle income context" or lmic or "low-middle income" or "low and middle income").mp.) |
| Limits | animals/ not humans.sh.  yr="2000 -Current" |

### EMBASE

| #1 | Asphyxia Neonatorum/ or Newborn hypoxia/ |
| --- | --- |
| #2 | "Birth asphyxia".mp. |
| #3 | #1 or #2 |
| #4 | ((Neonat* or newborn or infant* or intrapartum or intrapartum-related or perinatal or baby or babies) adj3 (encephalopath* or hypoxi* or asphyxia* or hypoxia-isch?emi# or hypoxic-isch?emi# or HIE or "therapeutic hypothermia" or "brain injury")).mp. 31915 |
| #5 | #3 or #4 |
| #6 | (outcome* or death* or mortalit* or morbidit* or surviv* or disabilit* or impair* or development* or neurodevelopment*).mp. |
| #7 | mortality/ or "cause of death"/ or child mortality/ or fatal outcome/ or hospital mortality/ or infant mortality/ or perinatal mortality/ or survival rate/ |
| #8 | death/ or brain death/ or infant death/ or perinatal death/ |
| #9 | #6 or #7 or #8 |
| #10 | (Predict* or risk* or factor* or prognos* or associat* or relat* or variable* or marker or diagnos* or detect* or identif* or sensitiv* or model* or score* or algorithm*).mp. [mp=title, abstract, heading word, drug trade name, original title, device manufacturer, drug manufacturer, device trade name, keyword heading word, floating subheading word, candidate term word] |
| #11 | #5 and #9 and #10 |
| #12 | (afghanistan or albania or algeria or angola or antigua or barbuda or argentina or armenia or armenian or aruba or azerbaijan or bahrain or bangladesh or barbados or belarus or byelarus or belorussia or byelorussian or belize or benin or dahomey or bhutan or bolivia or bosnia or herzegovina or botswana or bechuanaland or brazil or brasil or bulgaria or "burkina faso" or "burkina fasso" or "upper volta" or burundi or urundi or "cabo verde" or "cape verde" or cambodia or kampuchea or "khmer republic" or cameroon or cameron or cameroun or "central african republic" or "ubangi shari" or chad or chile or china or colombia or comoros or "comoro islands" or "iles comores" or mayotte or "drc" or congo or zaire or "costa rica" or "cote d'ivoire" or "coted' ivoire" or "cote divoire" or "cote d ivoire" or "ivory coast" or croatia or cuba or cyprus or "czech republic" or czechoslovakia or djibouti or somaliland or dominica or "dominican republic" or ecuador or egypt or "united arab republic" or "el Salvador" or "equatorial guinea" or guinea or eritrea or estonia or eswatini or swaziland or ethiopia or fiji or gabon or "gabonese republic" or gambia or georgia or ghana or gibraltar or greece or grenada or guam or guatemala or guinea or "guinea Bissau" or guyana or guiana or haiti or hispaniola or honduras or hungary or india or indonesia or timor or iran or iraq or jamaica or jordan or kazakhstan or kazakh or kenya or korea or kosovo or kyrgyzstan or kirghizia or kirgizstan or "kyrgyz republic" or kirghiz or laos or "lao pdr" or "lao people's democratic republic" or latvia or lebanon or lesotho or basutoland or liberia or libya or lithuania or macau or macao or macedonia or madagascaror malagasy republic or malawi or nyasaland or malaysia or "malay federation" or "malaya federation" or maldives or "indian ocean" or mali or malta or micronesia or kiribati or "marshall islands" or nauru or "northern mariana islands" or palau or tuvalu or mauritania or mauritius or mexico or moldova or mongolia or montenegro or morocco or ifni or mozambique or myanmar or burma or namibia or nepal or antilles or nicaragua or niger or nigeria or oman or muscat or pakistan or panama or "papua new guinea" or "new guinea" or paraguay or peru).mp. |
| #13 | (philippines or philipines or phillipines or phillippines or poland or "polish people's republic" or portugal or "portuguese republic" or "puerto rico" or romania or russia or "russian federation" or ussr or "soviet union" or "union of soviet socialist republics" or rwanda or ruanda or samoa or "pacific islands" or polynesia or "samoan islands" or "navigator island" or "navigator islands" or "sao tome and principe" or "saudi arabia" or senegal or serbia or seychelles or "sierra leone" or slovakia or "slovak republic" or slovenia or melanesia or "solomon island" or "solomon islands" or "norfolk island" or "norfolk islands" or somalia or "south Africa" or "south sudan" or "sri lanka" or ceylon or "saint kitts and nevis" or "st.kitts and nevis" or "saint lucia" or "st. lucia" or "saint vincent and the grenadines" or "saint Vincent" or "st. vincent" or grenadines or sudan or suriname or surinam or guiana or syria or "syrian arab republic" or tajikistan or tadjikistan or tadzhikistan or tadzhik or tanzania or tanganyika or thailand or siam or "timor leste" or "east timor" or togo or "togolese republic" or tonga or "trinidad and tobago" or "trinidad or Tobago" or tunisia or turkey or turkmenistan or turkmen or uganda or ukraine or uruguay or uzbekistan or uzbekor vanuatu or venezuela or vietnam or "west bank" or gaza or palestine or yemen or yugoslavia or zambia or zimbabwe or Rhodesia).mp.or #1 |
| #14 | ("sub-saharan" or subsaharan or "sub saharan" or Africa or sahara or "west indies" or caribbean or "south america" or "south asia" or "middle east" or "developing country" or "third world" or "under developed country" or "underdeveloped country" or "middle income country" or "middle income nation" or "low income country" or "low income nation" or "low income setting" or "low income context" or "middle income setting" or "middle income context" or lmic or "low-middle income" or "low and middle income").mp. |
| #15 | #12 or #13 or #14 |
| #16 | #11 and #15 |
| #17 | 16 not Animals |
| #18 | limit 17 to yr="2000-Current" |

### Cochrane Library

| #1 | MeSH descriptor: [Asphyxia Neonatorum] this term only |
| --- | --- |
| #2 | (Neonat* or newborn or infant* or intrapartum or birth or perinatal or baby) NEAR (encephalopath* or hypoxi* or asphyxia* or HIE or brain injur*) |
| #3 | #1 or #2 |
| #4 | (outcome* or death* or mortalit* or surviv* or disabilit* or impairment* or development* or neurodevelopment*) with Cochrane Library publication date Between Jan 2000 and Jul 2023 |
| #5 | MeSH descriptor: [Death] this term only |
| #6 | #4 or #5 |
| #7 | #3 and #6 with Cochrane Library publication date Between Jan 2000 and Jul 2023 |
| #8 | MeSH descriptor: [Asphyxia Neonatorum] this term only |
| #9 | (Neonat* or newborn or infant* or intrapartum or birth or perinatal or baby) NEAR (encephalopath* or hypoxi* or asphyxia* or HIE or brain injur*) |
| #10 | #8 or #9 |
| #11 | (outcome* or death* or mortalit* or surviv* or disabilit* or impairment* or development* or neurodevelopment*) with Cochrane Library publication date Between Jan 2000 and Jul 2023 |
| #12 | MeSH descriptor: [Death] this term only |
| #13 | #11 or #12 |
| #14 | #10 and #13 with Cochrane Library publication date Between Jan 2000 and Jul 2023 |
| #15 | afghanistan OR albania OR algeria OR american samoa OR angola OR antigua OR barbuda OR argentina OR armenia OR armenian OR aruba OR azerbaijan OR bahrain OR bangladesh OR barbados OR belarus OR byelarus OR belorussia OR byelorussian OR belize OR british honduras OR benin OR dahomey OR bhutan OR bolivia OR bosnia OR herzegovina OR botswana OR bechuanaland OR brazil OR brasil OR bulgaria OR burkina faso OR burkina fasso OR upper volta OR burundi OR urundi OR cabo verde OR cape verde OR cambodia OR kampuchea OR khmer republic OR cameroon OR cameron OR cameroun OR central african republic OR ubangi shari OR chad OR chile OR china OR colombia OR comoros OR Comoro islands OR mayotte OR congo OR zaire OR costa rica OR cote  d'ivoire OR cote d'ivoire OR cote d'ivoire OR ivory coast OR Croatia OR cuba OR cyprus OR czech republic OR czechoslovakia OR  djibouti OR french somaliland OR dominica OR dominican republic OR ecuador OR egypt OR united arab republic OR el salvador OR  equatorial guinea OR spanish guinea OR eritrea OR estonia OR eswatini OR swaziland OR ethiopia OR fiji OR gabon OR gabonese  republic OR gambia OR georgia OR georgian OR ghana OR gold coast OR gibraltar OR greece OR grenada OR guam OR guatemala  OR guinea OR guyana OR guiana OR haiti OR hispaniola OR honduras OR hungary OR india OR indonesia OR timor OR iran OR  iraq OR isle of man OR jamaica OR jordan OR kazakhstan OR kazakh OR kenya OR korea OR kosovo OR kyrgyzstan OR kirghizia  OR kirgizstan OR kyrgyz republic OR kirghiz OR laos OR lao pdr OR lao people's democratic republic OR latvia OR lebanon OR  lesotho OR basutoland OR liberia OR libya OR libyan arab jamahiriya OR lithuania OR macau OR macao OR macedonia OR  madagascar OR malagasy republic OR malawi OR nyasaland OR malaysia OR maldives OR indian ocean OR mali OR malta OR  micronesia OR kiribati OR marshall islands OR nauru OR northern mariana islands OR palau OR tuvalu OR mauritania OR mauritius  OR mexico OR moldova OR moldovian OR mongolia OR montenegro OR morocco OR ifni OR mozambique OR portuguese  east africa OR myanmar OR burma OR namibia OR nepal OR netherlands antilles OR nicaragua OR niger OR nigeria OR oman OR  muscat OR pakistan OR panama OR papua new guinea OR Paraguay OR peru OR philippines OR philipines OR phillipines OR  phillippines OR poland OR polish people's republic OR portugal OR portuguese republic OR puerto rico OR romania OR russia OR  russian federation OR ussr OR soviet union OR union of soviet socialist republics OR rwanda OR ruanda OR samoa OR pacific  islands OR polynesia OR samoan islands OR sao tome and principe OR saudi arabia OR senegal OR serbia OR seychelles OR sierra leone OR slovakia OR slovak republic OR slovenia OR melanesia OR solomon island OR solomon islands OR norfolk island OR Somalia OR south africa OR south sudan OR sri lanka OR ceylon OR saint kitts and nevis OR st kitts and nevis OR saint lucia OR st lucia OR saint vincent OR st vincent OR grenadines OR sudan OR suriname OR surinam OR syria OR syrian arab republic OR tajikistan OR tadjikistan OR tadzhikistan OR tadzhik OR tanzania OR Tanganyika OR thailand OR siam OR timor leste OR east timor OR togo OR  togolese republic OR tonga OR trinidad OR tobago OR tunisia OR turkey OR turkmenistan OR turkmen OR uganda OR ukraine OR  uruguay OR uzbekistan OR uzbek OR vanuatu OR new hebrides OR venezuela OR vietnam OR viet nam OR middle east OR west bank OR gaza OR palestine OR yemen OR yugoslavia OR zambia OR zimbabwe OR northern rhodesia OR global south OR africa south of the sahara OR sub saharan africa OR subsaharan africa OR central africa OR north africa OR northern africa OR magreb OR maghrib OR sahara OR southern africa OR east africa OR eastern africa OR west africa OR western africa OR west indies OR indian ocean islands OR caribbean OR central america OR latin america OR south america OR central asia OR north asia OR northern asia OR southeastern asia OR south eastern asia OR southeast asia OR south east asia OR western asia OR east europe OR eastern europe OR developing country OR developing countries OR developing nation OR developing nations OR developing population OR developing populations OR developing world OR less developed country OR less developed countries OR less developed nation OR less developed nations OR less developed world OR lesser developed countries OR lesser developed nations OR under developed country OR under developed countries OR under developed nations OR under developed world OR underdeveloped country OR underdeveloped countries OR underdeveloped nation OR underdeveloped nations OR underdeveloped population OR underdeveloped populations OR underdeveloped world OR middle income country OR middle income countries OR middle income nation OR middle income nations OR middle income population OR middle income populations OR low income country OR low income countries OR low income nation OR low income nations OR low income population OR low income populations OR lower income country OR lower income countries OR lower income nations OR lower income population OR lower income populations OR underserved countries OR underserved nations OR underserved population OR underserved populations OR under served population OR under served populations OR deprived countries OR deprived population OR deprived populations OR poor  country OR poor countries OR poor nation OR poor nations OR poor population OR poor populations OR poor world OR poorer countries OR poorer nations OR poorer population OR poorer populations OR developing economy OR developing economies OR less developed economy OR less developed economies OR underdeveloped economies OR middle income economy OR middle income economies OR low income economy OR low income economies OR lower income economies OR low gdp OR low gnp OR low gross domestic OR low gross national OR lower gdp OR lower gross domestic OR lmic OR lmics OR third world OR lami country OR lami countries OR transitional country OR transitional countries OR emerging economies OR emerging nation OR emerging nations) |
| #16 | #14 and #15 with Cochrane Library publication date Between Jan 2000 and Apr 2024, in Cochrane Reviews, Cochrane Protocols, Trials |

### Global Index Medicus

| #1 | tw:((tw:(tw:((tw:(neonat* OR newborn OR infant* OR intrapartum OR "intrapartum-related" OR perinatal OR baby OR babies)) AND (tw:("birth asphyxia" OR encephalopath* OR hypoxi* OR “hypoxic-ischemic” OR hypoxic-ischaemic” OR “hypoxia-ischaemia” OR "therapeutic hypothermia" OR “hypoxia-ischemia” OR asphyxia* OR hie OR "brain injury")) AND (tw:(outcome* OR death* OR mortalit* OR morbidit* OR surviv* OR disabilit* OR impair* OR development* OR neurodevelopment*)) AND (tw:(predict* OR risk* OR factor* OR prognos* OR associat* OR marker OR diagnos* OR detect* OR identif* OR valid* OR sensitiv* OR relat* OR variable* OR model* OR score* OR algorithm* OR sarnat OR thompson)) AND NOT (mh:(animal* OR sheep OR lamb OR rodent* OR rat* OR mouse OR mice)) ) AND (year_cluster:[2002 TO 2024])))) |
| --- | --- |

Supplementary material 3: Quality in Prognosis Studies (QUIPS) risk of bias scoring proforma


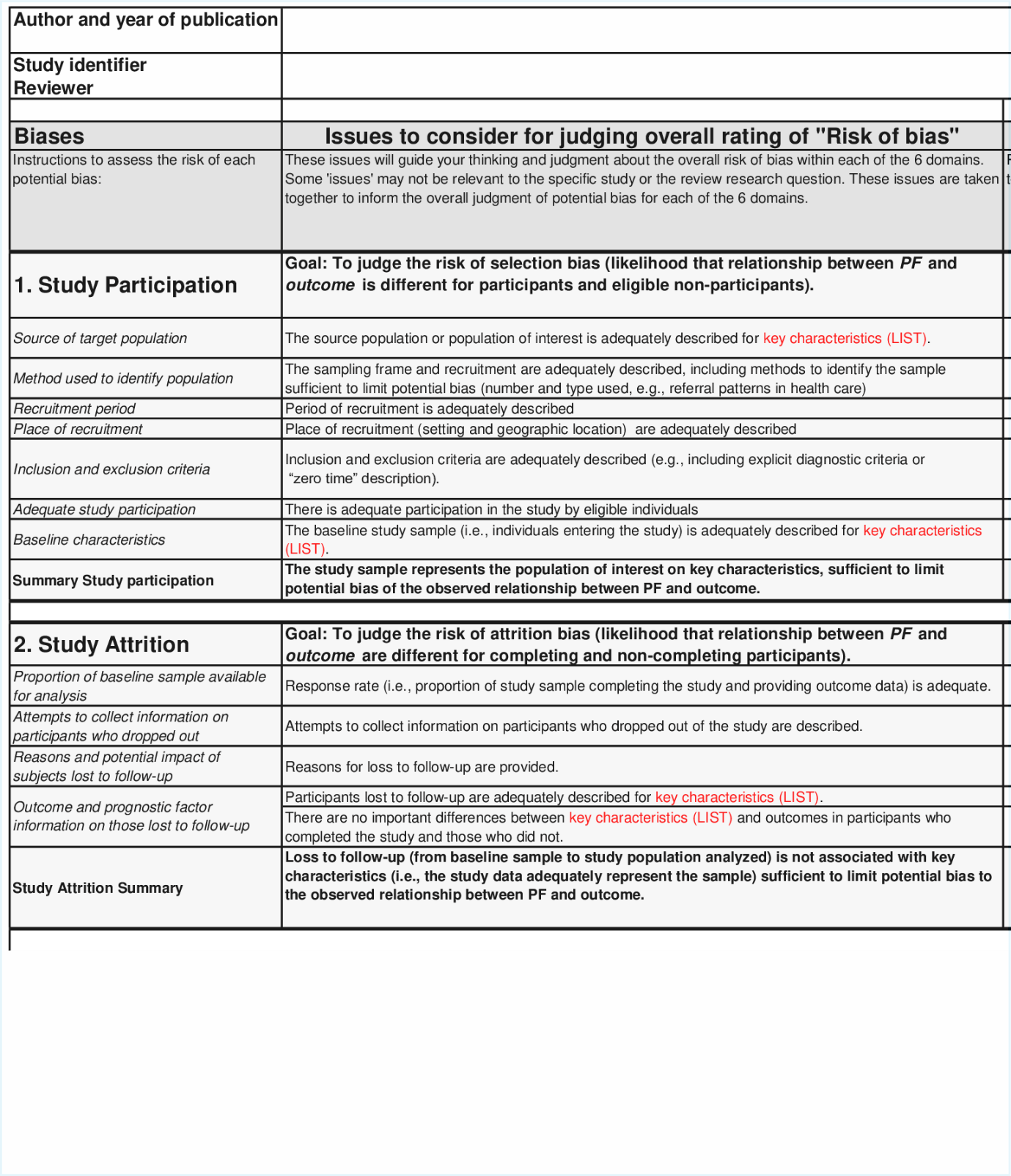


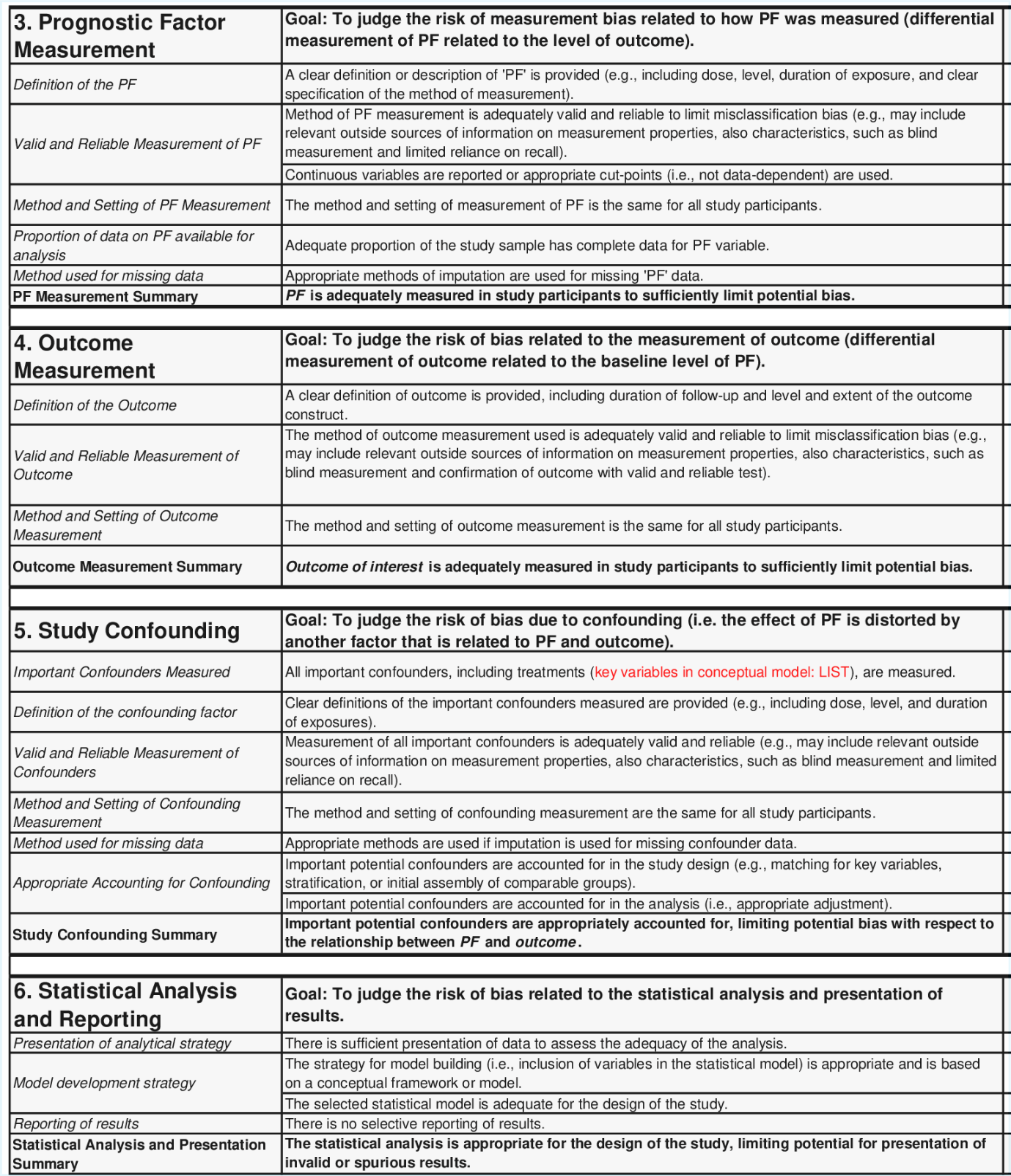


Each domain, between 3-7 items, is assigned one of four ratings (yes, partial, no, unsure). Based on the distribution of item ratings, an overall risk of bias (RoB) rating of low, moderate, or high is subsequently assigned to each domain.^1^ Finally, an overall RoB rating was assigned for the study: low RoB was defined as: all domains ‘low’, or up to one ‘moderate’; high RoB was defined as at least one domain rated as ‘high’, or ≥3 ‘moderate’; moderate RoB was defined as any article in between these definitions. We excluded the ‘confounding’ domain (no. 5) from the original tool, due to the understanding that confounding is a causal concept, and adjustment in predictive studies could lead to bias by overfitting the prediction.^2-3^

*References: [1] Hayden JA, van der Windt DA, Cartwright JL, Cote P, Bombardier C. Assessing bias in studies of prognostic factors. Ann Intern Med. 2013 Feb 19;158(4):280–6. [2] Schooling CM, Jones HE. Clarifying questions about “risk factors”: predictors versus explanation. Emerg Themes Epidemiol. 2018;15:10 [3] Hernán MA, Hsu J, Healy B. A second chance to get causal inference right: a classification of data science tasks. Chance. 2019;32(1):42–9.*

Supplementary material 4: Characteristics and predictor data reported in included articles

Ordered according to predictor modality (neonatal clinical assessment of NE severity, neurophysiology, neuroimaging, biomarker, postneonatal neurological assessment), and by year of publication.

| **First author, year** | **Study design** | **Country of study** | **Recruitment years** | **Neuro-protection(n)*** | **NE definition** | **Total no**;**  **No. with death/NDI** | **Outcome: duration of follow-up, definition** | **Predictor definition** | **Data for prediction of outcome reported** |
| --- | --- | --- | --- | --- | --- | --- | --- | --- | --- |
| ***a. Neonatal clinical assessments of NE severity*** | | | | | | | | | |
| Mathieson, 2024 ^1^ | PC | Uganda | 2019-20 | None | ≥36 wk;  Apgar <7 at 5 min/ prolonged resus at birth;  + Thompson ≥7 | 39;  Death: 14  NDI: 7 | 12-24m: Death or NDI- BSID-III cognitive/ motor (<70), HINE (<67), CP (GMFCS 3-5) | Thompson ≥7:  Day 1  Peak day 1-5 | For death/NDI :  Sens 75 (51-91), Spec 84 (60-97), PPV 83 (59-96), NPV 76 (53-92), AUC 0.79 (0.67-0.92)  Sens 85 (CI 62-97), Spec 58 (33-80), PPV 68 (46-85), NPV 79 (49-95), AUC 0.71 (0.58-0.85) |
| Tran, 2024 ^2^ | P | Vietnam | 2016-19 | TH | ≥36 wk, <6h old;  Apgar ≤5 or resus at 10 min  + Neurology abnormal (Sarnat);  + aEEG abnormal background or seizures | 112  Death: 43  NDI: 30 | 18m: Death or NDI- HINE, ASQ (>-2 SD below mean), CP (Bax criteria) | Sarnat III | Death/NDI vs. favourable outcome- n:  58/73 vs 26/39  TP=58, FP=26, FN=15, TN=13 |
|  |  |  |  |  |  | 69  NDI: 30 | 18m: NDI- HINE, ASQ (>-2 SD below mean), CP (Bax criteria) | Sarnat III | NDI vs no NDI- n:  23/30 vs 26/39  TP=23, FP=26, FN=7, TN=13 |
| Huang, 2022 ^3^ | PC | China | 2013-20 | None | ≥37 wk + 2.5–4kg;  1st artery pH <7/ Apgar ≤3 at 5 min;  + Neurology abnormal (Sarnat);  + ≥2 organ dysfunction shortly after birth | 50  NDI: 18 | 18m: NDI- BSID-II (MDI/PDI 70–79=mod, <70=severe), CP, visual/hearing loss | Sarnat:  I  II  III    II-III | Severe NDI vs mod NDI vs no-mild NDI- n (%):  4/13 (30.8%) vs 0/5 (0%) vs 11/32 (34.4%)  7/13 (53.9%) vs 4/5 (80.0%) vs 21/32 (65.6%)  2/13 (15.4%) vs 1/5 (20%) vs 0/32 (0%)  For mod-severe NDI: TP=3, FP=0, FN=15, TN=32  For mod-severe NDI: TP=14, FP=21, FN=4,TN=11 |
| Boskabadi, 2021 ^4^ | PC | Iran | 2013-17 | None | ≥2 of: a) Abnormal CTG/ fetal brady); b) Mec + hypotonia / bradycardia/ resp distress immediately after birth; c) Apgar <4 1min/ <7 5min; d) Resus >1min; e) pH <7.2 and BE >-12;  + Neurology abnormal (Sarnat) | 32  NDI: 25 | 24m: NDI-DDST II | Sarnat:  I  II  III  II-III | NDI vs no NDI- n (%):  3/25 (12.0%) vs 6/7 (85.7%)  10/25 (40.0%) vs 1/7 (14.3%)  12/25 (48.0%) vs 0/7 (0.0%) - TP=12, FP=0, FN=13, TN=7  TP=22, FP=1, FN=3, TN=6 |
| Montaldo, 2020 ^5^ | RCT | India, Sri Lanka, Bangladesh | 2016-19 | TH (50%) vs none | ≥36 wk, ≥1.8kg, <6h old;  Apgar ≤5/ resus/ no cry at 5 min  + Neurology abnormal (Sarnat) | 45  Death:22  NDI: 1 | 18-22m: Death, or NDI- BSID III (cognitive <85), CP, hearing/ visual loss | Sarnat III | Death/NDI vs favourable outcome- n, p-value:  9/23 vs 2/22, p=0.01 - TP=9, FP=2, FN=14, TN=20 |
| Preeti, 2019 ^6^ | PC | India | 2007-08 | None | Term;  Resus required;  + Neurology abnormal (Sarnat) | 76  Death: 28  NDI: 20 | 12m: Death or NDI- DAS-II (DQ ≤85), Amiel-Tison | Sarnat III | Death/NDI vs favourable outcome- n (%), p-value:  23/28 (82%) vs.4/48 (8.3%), p<0.001  TP=23, FP=4, FN=5, TN=44 |
|  |  |  |  |  |  | 43  NDI: 20 | 12m: NDI- DAS-II DQ ≤85, Amiel- Tison | Sarnat III | NDI vs no NDI- n, p-value:  4/20 vs. 0/23, p=0.04 - TP=4, FP=0, FN=16,TN=23 |
| Tann, 2018 ^7^ | PCC | Uganda | 2011-12 | None | ≥37 wk, <12h old;  Apgar ≤5 at 5 min/ resus at birth;  + Thompson ≥6 | 197  Death:81  NDI: 34 | 27-30m: Death, or NDI- GMDS-II (DQ <70), HINE (<67), CP (GMFCS 3-5) | Sarnat, peak day 1-5:  I  II  III  II-III  Thompson, peak day 1-5:  6-10  11-14  15+  11+ | Death/NDI vs favourable outcome, n, p-value:  p=<0.0001  5/115 vs 18/82  54/115 vs 52/82  56/115 vs 12/82 - TP=56, FP=12, FN=59, TN=70  TP=110, FP=64, FN=5, TN=18  p<0.0001  30/115 vs 50/82  66/115 vs 30/82  19/115 vs 2/82 - TP=19, FP=2, FN=96, TN=80  TP=85, FP=32, FN=30, TN=50 |
|  |  |  |  |  |  | 116  NDI: 34 | 27-30m: NDI- GMDS-II (DQ <70), HINE (<67), CP (GMFCS 3-5) | Sarnat, peak day 1-5:  I  II  III  II-III  Thompson, peak day 1-5:  6-10  11-14  15+  11+ | NDI vs no NDI- n; RR (95% CI):  p=0.04  2/34 vs 18/82  22/34 vs 52/82; RR 3.0 (CI 0.8-11.6)  10/34 vs 12/82; RR 4.6 (CI 1.1-18.3)  TP=10, FP=12, FN=24, TN=70  TP=32, FP=64, FN=2, TN=18  p=0.01  13/34 vs 50/82  16/34 vs 30/82; RR 6.3 (3.4-11.8)  5/34 vs 2/82; RR 13.0 (6.7-25.3)  TP=5, FP=2, FN=29, TN=80  TP=21, FP=32, FN=13, TN=50 |
| Malla, 2017 ^8^ | RCT | India | 2012-15 | EPO (50) vs. none | ≥37 wk;  Apgar <5 at 10 min;  ≥2 of: abnormal CTG or FHR or meconium; resus >10 min; BE >16/ pH ≤7.0 in 1st hr;  + Neurology abnormal (Sarnat) | 100  Death: 16  NDI: 39 | 18-22m: Death or NDI- BSID-II, seizures, CP (GMFCS 2-5), visual/ hearing loss | Sarnat:  II  III | Death/NDI vs favourable outcome, n:  21/55 vs 29/45  34/55 vs 16/45 - TP 34, FP 16, FN 21, TN 29 |
| Kali, 2016 ^9^ | RC | South Africa | 2008-11 | TH | ≥36wk, >1.8kg;  ≥1 of resus ≥10 min/ Apgar ≤7 at 10 min/ 1^st^ hr pH ≤7 or BE ≤-16;  + Neurology abnormal (Thompson ≥10/ seizures/ mod-severe NE) | 67  Death: 17  NDI: 9 | 12m: Death, or NDI- BSID III, Amiel-Tison, hearing/ vision loss | Thompson (peak day 1-7):  ≤10  11-14  15+  11+ | Death/NDI vs favourable outcome, n (%), p-value:  4/26 (15.4%) vs 21/41 (51.2%), p=0.004  8/26 (30.8%) vs 16/41 (39%), p=0.604  14/26 (53.8%) vs 4/41 (9.8%), p=0.0001  TP 14, FP 4, FN 12, TN 37  TP 22, FP 20, FN 4, TN 21 |
| Lally, 2014 ^10^ | PC | India | 2009 | TH  (17) vs none | ≥37 wk/ ≥1.8kg; <6h old;  Resus/ Apgar ≤5 at 5 min;  + Thompson ≥5 | 42  Death: 6  NDI: 15 | 3.5 years: Death or NDI- BSID III (cognitive <85/ motor <82), CP (GMFCS 1-5), visual loss, epilepsy, OFC (>2SD below mean | Sarnat, day 3:  I  II  III  II-III | Death/NDI vs favourable outcome- n:  8/21 vs 16/21  7/21 vs 5/21  6/21 vs 0/21- TP=6, FP=0, FN=15, TN=21  TP=13, FP=5, FN=8, TN=16 |
|  |  |  |  |  |  | 36  NDI: 15 | 3.5 years: NDI- BSID III (cognitive <85/ motor <82), CP (GMFCS 1-5), visual loss, epilepsy, OFC (>2SD below mean | Sarnat, day 3:  I  II  III  II-III | NDI vs no NDI- n:  8/15 vs 16/21  5/15 vs 5/21  2/15 vs 0/21- TP=2, FP=0, FN=13, TN=21  TP=7, FP=5, FN=8, TN=16 |
| Polat, 2013 ^11^ | RC | Turkey | 2006-08 | None | ≥37 wk;  Apgar <5 at 5 min, pH ≤7.0 day 1, resus, onset resp ≥5 min, abnormal FHR or mec;  + NE (Levene); seizures day 1; organ dysfunction (brain + ≥1 organ | 25  NDI: 6 | 12m: NDI- abnormal ‘neurological examination’/ DDST-II | Levene:  I  II  III  II-III | NDI vs no NDI- n, p-value:  p=0.08  1/6 vs 10/19  3/6 vs 7/19  2/6 vs 2/19 - TP=2, FP=2, FN=4, TN=17  Sens 83.3, Spec 52.6, PPV 90.9, NPV 35.7 |
| Ong, 2009 ^12^ | ?PC | Malaysia | 2000 - 01 | None | 37-42 wk;  Abnormal CTG/ fetal brady/ thick mec/ cord pH <7.2 or BE >-15/ Apgar <6 at 5min/ resus >1min;  + Neurology abnormal (Thompson) | 38  Death: 8  NDI: 5 | 44-48m: NDI- ‘mod-severe motor’, DDST (>30% age-normative values), post-neonatal seizures | Sarnat:  I  II  III  II-III  Thompson at 3-8h:  ≥15 | Death/NDI vs. favourable outcome- n (%):  5/13 (38.5%) vs. 21/25 (84%)  1/13 (7.7%) vs. 4/25 (16%)  7/13 (53.8%) vs. 0/15 (0%) - TP=7, FP=0, FN=6, TN=25  8/13 vs. 4/15  Sens 61.5, Spec 84, PPV 75, NPV 80.8  7/13 (53.8%) vs. 0/15 (0%)  Sens 53.8, Spec 100, PPV 100, NPV 80.6 |
| Khedr, 2009 ^13^ | PCC | Egypt | ? | None | >37 wk;  Abnormal FHR / mec;  Apgar 5min <7/ 1st art pH <7.20/ delayed resp >5min/ resus at birth;  + Neurology abnormal (Thompson) | 20    Death: 1  NDI: 7 | 12m: Death, or NDI- CP, severe motor dysfunction | Thompson:  <11  11-15  16+  11+ | Death/NDI vs favourable outcome, n:  0/6 vs. 6/14  2/6 vs. 8/14  4/6 vs. 0/14 - TP=4, FP=0, FN=2, TN=14  TP=6, FP=8, FN=0, TN=6 |
| El Ayouty, 2007 ^14^ | PC | Egypt | 2002-04 | None | ≥38 wk, <24h old;  Apgar ≤3 at 5 min/ delayed 1^st^ breath >5min, 1^st^ bicarb <12;  + Neurology abnormal (Sarnat) | 25  NDI: 18 | 18m: NDI- DDST II | Sarnat, day 1:  II-III  III | NDI vs no NDI- n:  18/18 vs 4/7 - TP=18, FP=4, FN=0, TN=3  3/18 vs 0/7 - TP=3, FP=0, FN=15, TN=7  Sens 17, Spec 100, PPV 100, NPV 31 |
| Belet, 2004 ^15^ | PC | Turkey | ? | None | >37 wk;  Abnormal CTG or mec/ Apgar ≤6 at 5 min/ resus at birth;  + Neurology abnormal (Sarnat) | 24  NDI: 15 | 3.5-4 years: NDI- CP, ‘developmental delay’, epilepsy | Sarnat:  I  II  III  II-III | NDI vs no NDI- n:  0/15 vs. 2/9  9/15 vs. 7/9  6/15 vs. 0/9 - TP=6, FP=0, FN=9, TN=9  TP=15, FP=7, FN=0, TN=2 |
| **First author, year** | **Study design** | **Country** | **Recruitment years** | **Neuro-protection (n)** | **NE definition** | **Total no*;**  **No. with death/NDI** | **Outcome: duration of follow-up, definition** | **Predictor** | **Statistical association with outcome reported** |
| ***b. Neurophysiology*** | | | | | | | | | |
| Mathieson, 2024^1^  ^[EL]^ | PC | Uganda | 2019-20 | None | ≥36 wk;  Apgar <7 at 5 min/ prolonged resus at birth;  + Thompson ≥7 | 39  Death: 13  NDI: 7 | 12-24m: Death or NDI- BSID-III cognitive/ motor (<70), HINE (<67), CP (GMFCS 3-5) | EEG (Murray criteria)-  Moderate-severe BG:    Highest day 1-5    At 12h    At 24h    At 48h    At 72h   Recovery by 72h  Severe BG:    Highest day 1-5    At 12h    At 24h    At 48h    At 72h | Death/NDI vs favourable outcome:  Sens 90, Spec 58, PPV 69, NPV 85, AUC 0.74  Sens 100, Spec 57.1, PPV 57.1, NPV 100, AUC 0.79  Sens 90, Spec 64.3, PPV  64.3, NPV 90, AUC 0.77  Sens 85.7, Spec 72.2, PPV 70.6, NPV 86.7, AUC 0.79  Sens 81.8, Spec 76.5, PPV  69.2, NPV 86.7, AUC 0.79  0/20 vs 3/19  To predict favourable outcome: TP=3, FP=0, FN=16, TN=20  Sens 94, Spec 68, PPV 64, NPV 98, AUC 0.85  Sens 100, Spec 71.4, PPV 66.7, NPV 100, AUC 0.86  Sens 90, Spec 78.6, PPV 75, NPV 91.7, AUC 0.84  Sens 71.4, Spec 88.9, PPV 83.3, NPV 80, AUC 0.80  Sens 63.6, Spec 100, PPV 100, NPV 81, AUC 0.82 |
| Tran, 2024 ^2^ | P | Vietnam | 2016-19 | TH | ≥36 wk, <6h old;  Apgar ≤5 or resus at 10 min  + Neurology abnormal (Sarnat);  + aEEG abnormal background or seizures | 112  Death: 43  NDI: 30 | 18m: Death, or NDI- HINE, ASQ (>-2 SD below mean), CP (Bax criteria) | aEEG <12h:  Severe BG (burst  suppression/ flat trace) | Death/NDI vs. favourable outcome, n:  57/65 vs 22/35  TP=57, FP=22, FN=8, TN=13 |
|  |  |  |  |  |  | 69  NDI: 30 | 18m: NDI- HINE, ASQ (>-2 SD below mean), CP (Bax criteria) | aEEG <12h:  Severe BG (burst  suppression/ flat trace) | NDI vs no NDI- n:  26/30 vs 22/35 - TP=26, FP=22, FN=4, TN=13 |
| Huang, 2022 ^3^ | PC | China | 2013-20 | None | ≥37 wk + 2.5–4kg;  1st art pH <7/ Apgar ≤3 at 5 min;  + Neurology abnormal (Sarnat);  + ≥2 organ dysfunction shortly after birth | 50  NDI: 18 | 18m: NDI- BSID-II (MDI/PDI 70–79=mod, <70=severe), CP, visual/hearing loss | aEEG <6h (Hellstrom-Westas criteria):  Any abnormality (BG  and/or seizures) | Severe NDI vs mod NDI vs no-mild NDI, n, p-value:  13/13 vs 5/5 vs 24/32, p<0.001  Sens 100, Spec 25, PPV 42.9, NPV 100  For mod-severe NDI: TP=18, FP=24, FN=0, TN=8 |
| Kali, 2016 ^9^ | RC | South Africa | 2008-11 | TH | ≥36 wk + >1.8kg;  ≥1 of resus ≥10 min/ Apgar ≤7 at 10 min/ 1^st^ hr pH ≤7 or BE ≤-16;  + Neurology abnormal (Thompson ≥10/ seizures/ mod-severe NE) | 67  Death: 17  NDI: 9 | 12m: Death, or NDI- BSID III, Amiel-Tison, hearing/vision loss | aEEG:  Severely abnormal BG  <72h  Recovery (from severely  abnormal BG <48h)  High seizure burden (>3 in  1h/ one >30 min) | Death/NDI vs favourable outcome, n (%), p-value:  17/26 (65.4%) vs. 6/41 (14.6%), p=0.0001 - TP=17, FP=6, FN=9, TN=35  3/17 (17.6%) vs. 4/6 (66.7%), p=0.045 - TP=3, FP=4, FN=14, TN=2  11/26 (42.3%) vs. 8/41 (19.5%), p=0.055 - TP=11, FP=8, FN=15, TN=33 |
| Jia, 2014 ^16^ | PC | China  [In Chinese] | 2012-13 | None | ≥37 wk;  Apgar ≤7 at 1 and 5 min/ cord art pH <7;  + Neurological dysfunction, and multiorgan injury | 83  NDI: 18 | 12m: NDI- CDCC Infant’s intelligence development test (MDI/PDI <70) | aEEG BG (Al Naqeeb criteria):  Severely abnormal <6h | NDI vs no NDI- n:  18/18 vs 0/65 - TP=18, FP=0, FN=0, TN=65 |
| Cseko 2013 ^17^ | RC | Hungary | 2005 - 09 | TH | ≥36 wk;  Resus ≥10 min/ Apgar score ≤5 at 5min/ pH <7.0/BE >-16 1st hr;  + ≥1 neuro sign (abnormal alertness, reflexes, hypotonia, seizures) | 70  Death: 19  NDI: 7 | 18-24m: Death, or NDI- BSID-II (PDI/MDI <85) | aEEG (Hellström-Westas criteria):  Abnormal BG, at:    6h    12h      18h    24h    36h    48h    60h  Seizures | Death/NDI vs favourable outcome- Sens, Spec, PPV, NPV (95% CI):  Sens 100 (CI 87-100), Spec 39.5 (25-56), PPV 50 (36-64), NPV 100 (81-100)  Sens 95.7 (78-100), Spec 55 (39-71), PPV 55 (39-71), NPV 95.7 (78-100)  Sens 95.7 (78-100), Spec 69.1 (53-82), PPV 62.9 (45-79), NPV 96.7 (83-100)  Sens 95.2 (77-100), Spec 73.8 (58-86), PPV 64.5 (45-81), NPV 96.9 (84-100)  Sens 94.7 (74-100), Spec 82.9 (68-93), PPV 72 (51-88), NPV 97.1 (85-100)  Sens 82.4 (57-96), Spec 92.7 (80-99), PPV 82.4 (57-96), NPV 92.7 (80-99)  Sens 80 (52-96), Spec 97.5 (87-100), PPV 92.3 (64-100), NPV 92.9 (81-99)  14/26 vs 24/44 - TP=14, FP=24, FN=12, TN=20 |
| Jose, 2013 ^18^ | P | India | 2010- 11 | None | ≥37wk;  ≥2 of: fetal brady, thick mec, abnormal CTG, cord art pH <7.2 or BE >-15, Apgar <6 at 5 min/ resus >10 min;  + Neurology abnormal (Sarnat) | 30  NDI: 15 | 12m: NDI- abnormal ‘neurological examination’/ DDST-II | EEG day 1-3 (Sefton criteria):  Permanent discontinuity/  burst suppression | NDI vs no NDI- n, p-value:  13/15 vs 0/15, p<0.001 - Sens 100, Spec 40, PPV 59.1, NPV 100 |
| Polat, 2013 ^11^ | RC | Turkey | 2006 - 08 | None | ≥37wk;  Apgar <5 at 5 min, pH ≤7.0 day 1, resus, onset resp ≥5min, abnormal FHR or mec;  + NE (Levene); seizures day 1; multiple organ dysfunction (brain + at least 1 organ) | 25  NDI: 6 | 44-48m: NDI- ‘mod-severe motor; DDST (>30% age-normative values), post-neonatal seizures | aEEG BG 48-72h (Laroia criteria):  Normal  Mild  Moderate  Severe  Mod-severe  Any abnormality | NDI vs no NDI- n, p-value:  p<0.001  0/6 vs 13/19  0/6 vs 6/19  3/6 vs 0/19  3/6 vs 0/19- TP=3, FP=0, FN=3, TN=19  TP=6, FP=0, FN=0, TN=19  Sens 100, Spec 68.4, PPV 50, NPV 100 |
| Ong, 2009 ^12^ | ?PC | Malaysia | 2000 - 01 | None | 37-42 wk;  Abnormal CTG/ fetal brady/ thick mec/ cord pH <7.2 or BE >-15/ Apgar <6 at 5min/ Resus >1min;  + Neurology abnormal (Thompson) | 38  Death: 8  NDI: 5 | 12m: Death, or NDI – BSID-II (MDI <69), CP, severe hearing loss/ visual loss | aEEG BG 3-8h (Sefton criteria):  Normal  Mild  Moderate  Severe  Mod-severe | Death/NDI vs favourable outcome, n (%):  p<0.001  1/13 (7.7%) vs. 12/25 (48%)  3/13 (23.1%) vs. 11/25 (44%)  2/13 (15.4%) vs. 2/25 (8%)  7/13 (53.8%) vs. 0/25 - Sens 53.8, Spec 100, PPV 100, NPV 80.6  9/13 vs 2/25- TP=9, FP=2, FN=4, TN=23 |
| Khedr, 2009 ^13^ | ? | Egypt | ? | None | >37 wk;  Abnormal FHR/mec;  Apgar 1min <7/ 1st art pH <7.20/ delayed resp >5min; Resus at birth;  + Thompson | 20  Death: 1  NDI: 5 | 12m: Death, or NDI- CP, severe motor dysfunction | Evoked potentials abnormal, 0-7 days:  VEP  ABR  SSEP | Death/NDI vs favourable outcome, n:  3/6 vs 0/14- TP=3, FP=0, FN=3, TN=14  5/6 vs 6/14- TP=5, FP=6, FN=1, TN=8  5/6 vs 2/14 - TP=5, FP=2, FN=1, TN=12 |
| El Ayouty, 2007 ^14^ | PC | Egypt | 2002-04 | None | ≥38 wk, <24h old;  Apgar ≤3 at 5 min/ delayed 1^st^ breath >5min, 1^st^ bicarb <12;  + Neurology abnormal (Sarnat) | 25  NDI: 18 | 18m: NDI- DDST II | aEEG BG <72h (Biagioni criteria):  Severe  Mod-severe | NDI vs no NDI- n:  15/18 vs 0/7 - TP=15, FP=0, FN=3, TN=7  18/18 vs 0/7- Sens 100, Spec 100, PPV 100, NPV 100 |
| Liu, 2007 ^19^ | PC | China  [In Chinese] | 2003-05 | None | Term;  Fetal brady/ intrapartum event;  Apgar ≤3 at 1 min and ≤5 at 5 min/cord arterial pH ≤7;  + Neurology abnormal | 25  Death: 5  NDI: 1 | 18m: Death, or NDI - BSID (MDI/PDI <70) | aEEG BG <6h (Liu criteria):  Any abnormality  Severe | Death/NDI vs favourable outcome, n :  6/6 vs 4/19 – TP=6, FP=4, FN=0, TN=15  6/6 vs 1/19 – TP=6, FP=1, FN=0, TN=18 |
| **First author, year** | **Study design** | **Country** | **Recruitment years** | **Neuro-protection (n)** | **NE definition** | **Total no*;**  **No. with death/NDI** | **Outcome: duration of follow-up, definition** | **Predictor** | **Statistical association with outcome reported** |
| ***c. Neuroimaging*** | | | | | | | | | |
| Nanyunja, 2024 ^20^ | PC  (Conference abstract) | Uganda | 2019-20 | None | ≥36 wk;  Apgar <7 at 5 min/ prolonged resus at birth;  + Thompson ≥7 | MRI=21  Death: 1  NDI: 5  MRS=19  Death: 1  NDI: 5 | 12-24m: Death, or NDI- BSID-III (cognitive/ motor <70), HINE (<67), CP (GMFCS 3-5) | MRI day 10:  Rutherford/Thoresen total  score ≥7  Rutherford mod-severe  changes (mod-severe  BGT, absent PLIC, and/or  severe WM)  NICHD score 2A/2B/3  (any BGT, ALIC, PLIC  or watershed infarction)  MRS day 10:  Lactate/NAA ≥0.15  NAA/Creatine ≤1.34  NAA/Choline ≤2.46 | Sens 83.3, Spec 92.3, PPV 83.3, NPV 92.3, AUC 0.88  Sens 83.3, Spec 92.3, PPV 83.3, NPV 92.3, AUC 0.88  Sens 100, Spec 73, PPV 60, NPV 100, AUC 0.87  Sens 83.3, Spec 92.3, PPV 83.3, NPV 92.3, AUC 0.88  Sens 83.3, Spec 92.3, PPV 83.3, NPV 92.3, AUC 0.88  Sens 83.3, Spec 84.6, PPV 71.4, NPV 91.7, AUC 0.84 |
| Tran, 2024 ^2^ | PC | Vietnam | 2016-19 | TH | ≥36 wk, <6h old;  Apgar ≤5 or resus at 10 min  + Neurology abnormal (Sarnat);  + aEEG abnormal background or seizures | 101  Death:35  NDI:30 | 18m: Death, or NDI- HINE, ASQ (>-2 SD below mean), CP (Bax criteria) | MRI day 7-10:  Abnormal (BGT,PLIC,  and/or diffuse WM)  BGT/PLIC abnormal  WM diffuse injury | Death/NDI vs. favourable outcome, n:  29/65 vs 8/36 - TP=29, FP=8, FN=36, TN=28  12/65 vs 3/36 - TP=12, FP=3, FN=53, TN=33  17/65 vs 5/36 - TP=17, FP=5, FN=48, TN=31 |
|  |  |  |  |  |  | 66    NDI: 30 | 18m: NDI- HINE, ASQ (>-2 SD below mean), CP (Bax criteria) | MRI day 7-10:  Abnormal (BGT,PLIC,  and/or diffuse WM)  BGT/PLIC abnormal  WM diffuse injury | NDI vs no NDI- n:  22/30 vs 8/36 - TP=22, FP=8, FN=8, TN=28  9/30 vs 3/36- TP=9, FP=3, FN=21, TN=33  13/30 vs 5/36 - TP=13, FP=5, FN=17, TN=31 |
| Huang, 2022 ^3^ | PC | China | 2013-20 | None | ≥37 wk, 2.5–4kg;  1st artery pH <7/ Apgar ≤3 at 5 min;  + Neurology abnormal (Sarnat);  + ≥2 organ dysfunction shortly after birth | 50  NDI: 18 | 18m: NDI- BSID-II (MDI/PDI 70–79=mod, <70=severe), CP, visual/hearing loss | MRI day 4-7 (Barkovich score):  0- BGT and cortex normal  1- BGT abnormal  2- Cortex abnormal  3- BGT and cortex  abnormal  4- Abnormal signal in entire cortex and basal nuclei  Grade 3-4 abnormality  Any BGT abnormality  Any of above | NDI vs no NDI- n (%), p-value:  p=0.063  1/18 (20%) vs 23/32 (71.9%)  0/18 (0%) vs 7/32 (21.9%)  4/18 (30.8%) vs 2/32 (6.3%)  6/18 (70.8%) vs 0/32 (0%)  7/18 (78.5%) vs 0/32 (0%)  13/18 vs 0/32 - TP=13, FP=0, FN=5, TN=32  13/18 vs 7/32 - TP=13, FP=7, FN=5, TN=25  Sens 94.4, Spec 71.9, PPV 65.4,NPV 95.8 |
| Aker, 2021 ^21^ | RCT | India | 2013-15 | TH (50%) vs none | ≥35 wk, BW >1.8kg, <6h old;  Apgar ≤5 or resus at 10 min/ cord or 1^st^ hr pH <7.00 or BE ≥-12;  + Neurology abnormal (NICHD) | 38  Death: 4  NDI: 9 | 18m: Death, or NDI- BSID-III (cognitive/ motor <85), CP (GMFCS 3-5), vision/ hearing loss, seizures | MRI day 4-6 (Rutherford score):  Moderate-severe changes  (mod- severe BGT, absent  PLIC, and/or severe WM)  BGT mod-severe  PLIC absent      PLIC absent/equivocal | Death/NDI vs favourable outcome:  8/13 vs 3/25 - TP=8, FP=3, FN=5, TN=22  Sens 61.5, (CI 31.6-86.1), Spec 90.0 (73.5-97.9), PPV 72.7 (45.6-89.5), NPV 84.4 (72.9-91.6), accuracy 81.4 (66.6-91.6)  Sens 61.5 (31.6-86.1), Spec 96.7 (82.2-99.9), PPV 88.9 (52.6-98.3), NPV 85.3 (74.4-92.1), accuracy 86.1 (72.1-94.7)  Sens 46.2 (19.2-74.9), Spec 100 (88.4-100.0), PPV 100 , NPV 81.1 (72.2-87.6), accuracy 83.7 (69.3-93.2)  Sens 76.9 (46.2-95.0), Spec 93.3 (77.9-99.2), PPV 83.3 (55.9-95.2), NPV 90.3 (77.5-96.1), accuracy 88.4 (74.9-96.1) |
| Apaydin, 2021 ^22^ | RC | Turkey | 2014-18 | TH (34), head cooling (13) | >37 wk;  Apgar <5 at 5 min/ abnormal FHR/ mec/ pH <7;  + Neurology abnormal (Sarnat) | 47  NDI: 9 | 24m: NDI- BSID-II (PDI/MDI <85), CP | MRI day 7-14:  Any BGT + severe WM  Any BGT + mild-mod WM  Any BGT + any WM  Any abnormalities (apart  from only mild WM) | NDI vs no NDI- n:  2/9 vs 0/38  2/9 vs 0/38  TP=4, FP=0 FN=5 TN=38  6/9 vs 2/38 - TP=6, FP=2 FN=3 TN=36  For BSID-II PDI: Sens 97, Spec 100, AUC 1.00 (CI 1-1), p<0.001  For BSID-II MDI: Sens 95, Spec 85.7, AUC 0.90 (CI 0.72-1.00), p<0.001  For CP: Sens 83.3, Spec 95, AUC 0.89 (CI 0.71-1.00), p=0.002 |
| El Beheiry, 2019 ^23^ | PC | Egypt | 2015 - 16 | TH | ≥37 wk;  Hx of perinatal asphyxia event; + Neurology abnormal (Sarnat) | 33  NDI: 16 | 12m: NDI- BSID-III (motor/ cognitive/ language <85), CP | MRI day 10-14:  Abnormal (including BGT,  PLIC, brainstem, cortex,  WM)  DTI- FA values:  Centrum semiovale,  ≤0.235  PLIC, ≤0.435  Occipital WM  Optic radiation  Genu of corpus callosum,  ≤0.45  Splenium, of corpus  callosum ≤0.45 | NDI vs no NDI- n:  9/16 vs 0/17  Sens 56.25, Spec 100, PPV 100, NPV 70.83, accuracy 78.79, p<0.001  Sens 93.8 (69.8–99.8), Spec 94.1 (71.3–99.9), PPV 93.7, NPV 94.1, AUC 0.949 (0.811–0.995), p<0.0001  Sens 100 (79.4–100), Spec 100 (80.5–100), PPV 100, NPV 100, AUC 1.00 (0.89–1.00), p<0.0001  AUC 0.58 (0.40–0.75), p=0.43  AUC 0.51 (0.33–0.69), p=0.92  Sens 56.3 (29.9–80.2), Spec 100 (80.5–100), PPV 100, NPV 78, AUC 0.81 (0.64–0.93), p<0.0001  Sens 60 (32.3–83.7), Spec 100 (80.5–100), PPV 100, NPV 73.9, AUC 0.81 (0.64–0.93), p<0.0001 |
| Tann, 2018 ^7^ | PCC | Uganda | 2011-12 | None | ≥37 wk, <12h old;  Apgar ≤5 at 5 min/ resus at birth;  + Thompson ≥6 | Day 1:  103  NDI: 34  Day 4-5:  81  NDI: 33 | 27-30m: NDI- GMDS-II (DQ <70), HINE (<67), CP (GMFCS 3-5) | Cranial US, mod-severe  (focal bilateral BG/ diffuse  mod-severe WM):  Day 1  Day 4-5 | NDI vs no NDI- n, RR (95% CI), p-value:  5/34 vs 8/69, RR 1.2 (0.6–2.5), p=0.66  TP=5, FP=8, FN=29, TN=61  28/33 vs 8/48, RR 7.0 (3.8–16.3), p<0.001  TP=28, FP=8, FN=5, TN=74 |
|  |  |  |  |  |  | 93  Death: 12  NDI: 33 | 27-30m: Death, or NDI- GMDS-II (DQ <70), HINE (<67), CP (GMFCS 3-5) | Cranial US, mod-severe  (focal bilateral BG/ diffuse  mod-severe WM):  Day 4-5 | Death/NDI vs favourable outcome, n, RR (CI):  39/45 vs 8/48 RR 6.4 (3.0, 13.6)  TP=39, FP=8, FN=6, TN=40 |
| Kali, 2016 ^9^ | RC | South Africa | 2008-11 | TH | ≥36wk, >1.8kg;  ≥1 of resus ≥10 min/ Apgar ≤7 at 10 min/ 1^st^ hr pH ≤7 or BE ≤-16;  + Neurology abnormal (Thompson ≥10/ seizures/ mod-severe NE) | 67  Death: 17  NDI: 9 | 12m: Death, or NDI- BSID III, Amiel-Tison, hearing/ vision loss | MRI, median day 115 (range  4-150 days) (Rutherford):  Any abnormality    Cranial US, median day 1  (range 1-5):  RI <0.55 | Death/NDI vs favourable outcome, n (%), p-value:  4/5 (20%) vs. 1/20 (5%); p= 0.002 - TP=4, FP=1, FN=1, TN=19  Death/NDI vs favourable outcome, n (%), p-value:  2/19 (10.5%) vs. 3/46 (6.5%); p=0.625  TP=2, FP=3, FN=17, TN=43 |
| Lally, 2014 ^10^ | PC | India | 2009 | TH (17) vs none | ≥37 wk/ ≥1.8kg; <6h old;  Resus at birth/ Apgar ≤5 at 5 min;  + Thompson ≥5 | 38  NDI: 16 | 3.5 years: NDI- BSID III (cognitive <85/ motor <82), CP (GMFCS 1-5), visual loss, epilepsy, OFC (>2 SD below mean) | MRI, mean day 9 (SD 3.6)  (Rutherford score):  Moderate-severe BGT  Low PLIC signal    Severe WM  Any of above mod-severe  abnormalities | NDI vs no NDI:  7/16 vs 3/22 - TP=7, FP=3, FN=9, TN=19  Sens 43 (CI 16-75), Spec 86 (69-94)  7/16 vs 1/22 - TP=7, FP=1, FN=9, TN=21  Sens 43 (CI 16-75), Spec 96 (82-99)  7/16 vs 2/22 - TP=7, FP=2, FN=9, TN=20  Sens 43 (CI 16-75), Spec 89 (73-96)  9/16 vs 5/22 - TP=9, FP=5, FN=7, TN=17  Sens 57 (CI 25-84), Spec 79 (61-90) |
| Jose, 2013 ^18^ | PC | India | 2010- 11 | None | ≥37 wk;  ≥2 of: fetal brady, thick mec, abnormal CTG, cord art pH <7.2 or BE >-15, Apgar <6 at 5 min/ resus >10 min;  + Neurology abnormal (Sarnat) | CT:  30  NDI: 15  MRI:  26  NDI :11 | 12m: NDI- abnormal ‘neurological examination’/ DDST-II | CT brain day 4-7:  Any abnormality (cerebral  oedema, hypodensities,  bleeds)  MRI brain at 10-12 wk  (Barkovich score):  0- BGT + cortex normal  1- BGT abnormal  2- Cortex abnormal  3- BGT + cortex abnormal  4- Entire basal nuclei +  cortex  Any BGT abnormality  Any of above abnormalities | NDI vs no NDI, n:  15/15 vs. 1/15  Sens 100, Spec 93.3, PPV 93.8, NPV 100, accuracy 96.7  NDI vs no NDI, n (%), p-value:  2/11 vs 14/15, p<0.001  2/11 (18.2%) vs. 0/15, p=0.169  1/11 (9.1%) vs 1/15 (6.7%), p=1.000  6/11 (54.5%) vs. 0/15, p=0.002  0/11 vs 0/15  8/11 vs 0/15 - TP=8, FP=0, FN=3, TN=15  9/11 vs 1/15 - Sens 81.8, Spec 93.3, PPV 90, NPV 87.5, accuracy 88.5 |
| Polat, 2013 ^11^ | RC | Turkey | 2006-08 | None | ≥37 wk;  Apgar <5 at 5 min, pH ≤7.0 day 1, resus, onset resp ≥5 min, abnormal FHR or mec;  +NE (Levene); seizures day 1; multiple organ dysfunction (brain + at least 1 organ) | 25  NDI: 6 | 44-48m: NDI- ‘mod-severe motor’, DDST (>30% age-normative values), post-neonatal seizures | MRI day 7-14:  Normal  Mild-mod (cortex and  parasagittal zones)  Severe (brainstem, ventral  cerebellar vermis, BGT,  perirolandic regions)  Any abnormality | NDI vs no NDI- n: p=0.01  1/6 vs. 11/19  2/6 vs. 7/19  3/6 vs. 1/19 - TP=3, FP=1, FN=3, TN=18  5/6 vs 8/19 - Sens 83.3, Spec 57.9, PPV 38.5, NPV 91.6 |
| Kalay, 2011 ^24^ | RC | Turkey | 2006- 10 | None | ≥35 wk;  ≥1 of Apgar <5 at 5 min/ BE >-16 1st hr/ resp onset >5 min/ c-section for fetal distress; resus at birth;  + Neurology abnormal (Sarnat);  + ≥1 organ involved in addition to brain | 21  10 | 1-4.5 yr: NDI- ADSI (general development age younger than 30 chronological age) | MRI <48h:  “Appearances compatible  with hypoxia in axial T1A,  T2A, FLAIR”  DWI <48h:  “Diffusion limitation in  favour of hypoxia on  echoplanar imaging+ADC” | NDI vs no NDI- n:  8/10 vs 5/11  Sens 80, Spec 54, PPV 61.5, NPV 75  7/10 vs 3/11  Sens 70, Spec 72.7, PPV 70, NPV 72.7 |
| Ong, 2009 ^12^ | ?PC | Malaysia | 2000 - 01 | None | 37-42 wk;  Abnormal CTG/ fetal brady/ thick mec/ cord pH <7.2 or BE >-15/ Apgar <6 at 5min/ resus at birth >1min;  + Neurology abnormal (Thompson) | 38  Death: 8  NDI: 5 | 44-48m: NDI- ‘mod-severe motor’, DDST (>30 age-normative values), post-neonatal seizures | Cranial US 3-8h:  Abnormal (focal or diffuse  echogenicities/ ventricular  dilatation,) | Death/NDI vs. favourable outcome, n (%), p value:  12/13 (92.3%) vs. 10/25 (40%), p=0.002  Sens 92.3, Spec 60, PPV 54.5, NPV 93.8 |
| El Ayouty, 2007 ^14^ | PC | Egypt | 2002-04 | None | ≥38 wk, <24h old;  Apgar ≤3 at 5 min/ delayed 1^st^ breath >5min, 1^st^ bicarb <12;  + Neurology abnormal (Sarnat) | 25  NDI: 18 | 18m: NDI- DDST II | MRI 1-4 weeks:  Any BGT  Mod-severe BGT  Severe WM  Mod-severe BGT/Severe  WM  Any abnormality | NDI vs no NDI- n:  10/18 vs. 3/7 - TP=10, FP=3, FN=8, TN=4  10/18 vs. 0/7 - TP=10, FP=0, FN=8, TN=7  6/18 vs. 0/7 - TP=6, FP=0, FN=12, TN=7  14/18 vs. 0/7 - TP=14, FP=0, FN=4, TN=7  18/18 vs. 4/7  Sens 100, Spec 43, PPV 82, NPV 100 |
| Belet, 2004 ^15^ | PC | Turkey | ? | None | >37 wk;  Fetal distress (abnormal CTG, mec)/ Apgar ≤6 at 5min/ resus at birth;  + Neurology abnormal (Sarnat) | 24  NDI: 15 | 3.5-4 yr: NDI- CP, ‘ developmental delay’, epilepsy | MRI abnormal (WM, grey matter, encephalomalacia,  atrophy):  Day 11  4 months | NDI vs. no NDI:  Sens 100, Spec 44, PPV 70.6, NPV 100  Sens 86.7, Spec 100, PPV 100, NPV 81.8 |
| **Author, year** | **Study design** | **Country** | **Recruitment years** | **Neuro-protection (n)** | **NE definition** | **Total no*;**  **No. with death/NDI** | **Outcome: duration of follow-up, definition** | **Predictor** | **Statistical association with outcome** |
| ***d. Biochemical markers*** | | | | | | | | | |
| Mfingwana, 2023 ^25^ | RC | South Africa | 2008-11 | TH | ≥36 wk;  Apgar <7 at 10 min/ resus >10 min/ acidosis 1st hr;  + Neurology abnormal (Thompson) | 60  Death: 9  NDI: 18 | 12m: Death, or NDI-BSID-III, Amiel-Tison, CP, vision/hearing loss | Nucleated RBCs, neonatal blood at <6h:  ≥30 cells/100 WBCs | Death/NDI vs favourable outcome, n:  8/27 vs 0/32 - TP=8, FP=0, FN=19, TN=32 |
|  |  |  |  |  |  | 50  NDI:18 | 12m: NDI- BSID-III, Amiel-Tison, CP, vision/ hearing loss | Nucleated RBCs, neonatal blood at <6h:  ≥30 cells/100 WBCs | NDI vs no NDI, n, p-value:  4/18 vs 0/32, p=0.013 - TP=4, FP=0, FN=14, TN=32 |
| Huang, 2022 ^3^ | PC | China | 2013-20 | None | ≥37 wk, 2.5–4kg;  1st art pH <7/ Apgar ≤3 at 5 min;  + Neurology abnormal (Sarnat);  + ≥2 organ dysfunction | 50  NDI: 18 | 18m: NDI- BSID-II (MDI/PDI 70–79=mod, <70=severe), CP, visual/ hearing loss | pH, 1^st^ arterial: <7.0  NSE, day 3: ≥27.3μg/L | NDI vs no NDI- n, p value :  7/18 vs 13/32, p=0.921  Sens 100, Spec 87.5, PPV 81.8, NPV 100 |
| Pang, 2021 ^26^ | PCC | Uganda | 2011-12 | None | ≥37 wk, <12h old;  Apgar ≤5 at 5 min/ resus at birth;  + Thompson ≥6 | 150  Death: 62  NDI: 27 | 27-30m: Death, or NDI- GMDS-II (DQ <70), HINE (<67), CP (GMFCS 3-5) | IL-10 (log10 value 0.225),  <12h | Sens 88.8, Spec 36.1, PPV 66.9, NPV 82.4, AUC 0.65 (CI 0.56–0.74) |
| Montaldo, 2020 ^5^ | RCT | India, Sri Lanka, Bangladesh | 2016-19 | TH | ≥36 wk, ≥1.8kg, <6h;  Apgar ≤5/ resus/ no cry at 5 min  + Neurology abnormal (Sarnat) | 45  Death: 22  NDI: 1 | 18-22m: Death, or NDI- BSID-III (cognitive <85), CP, hearing/ visual loss | Coagulation ‘abnormality’ | Death/NDI vs favourable outcome, n (%), p-value:  11/23 (47.8%) vs 3/22 (13.6%), p=0.02 |
| Preeti, 2019 ^6^ | PC | India | 2007-08 | None | Term;  Resus required;  + Neurology abnormal (Sarnat) | 72  Death: 8  NDI: 20 | 12m: Death, or NDI- DAS-II (DQ ≤85) | Arterial blood gas (1^st^):  pH ≤7.1  BE >-16 | Death/NDI vs. favourable outcome, n (%), p value:  20/28 vs. 10/44, p<0.001 - TP=20, FP=10, FN=8, TN=34  22/28 vs. 18/44 , p=0.003 - TP=22, FP=18, FN=6, TN=26 |
|  |  |  |  |  |  | 43  NDI: 20 | 12m: NDI- DAS-II (DQ ≤85) | Arterial blood gas (1^st^):  pH ≤7.1  BE >-16 | NDI vs no NDI, n (%), p value:  16/20 vs. 1/23, p<0.0001  TP=16, FP=1, FN=4, TN=22  17/20 vs. 2/23, p<0.0001  TP=17, FP=2, FN=3, TN=21 |
| Kali, 2016 ^9^ | RC | South Africa | 2008-11 | TH | ≥36wk, >1.8kg;  ≥1 of resus ≥10 min/ Apgar ≤7 at 10 min/ 1^st^ hr pH ≤7.0 or BE ≤-16;  + Neurology abnormal (Thompson ≥10/ seizures/ mod-severe NE) | 67  Death: 17  NDI: 9 | 12m: Death, or NDI- BSID III, Amiel-Tison, hearing/ vision loss | Coagulation ‘abnormality’  ‘Thrombocytopenia’ | Death/NDI vs favourable outcome, n (%), p value:  9/26 (34.6%) vs 12/41 (29.3%), p=0.788  TP=9, FP=12,FN=17, TN=29  9/26 (34.6%) vs 6/41 (14.6%), p=0.074  TP=9, FP=6, FN=17, TN=35 |
| Gane, 2014 ^27^ | RCT | India | 2011-13 | TH (53) vs none | ≥37 wk;  Art pH <7.0 in 1st hr/ BE >-16;  ≥2 of: Apgar 10 min <5, fetal distress; resus ≥10 min after birth, evidence of any organ dysfunction;  + NE (NICHD) | 103  Death: 12  NDI: 23 | 12m: Death, or NDI- DAS-II (DQ <70) | At birth and 36h:  Blood ‘olive tail moment’-  12.85 arbitrary units  Serum 8-OHdG - 685.5  pg/ml | Sens 84.6, Spec 85.4, AUC 0.89 (CI 0.81-0.98) , p<0.001  Sens 80.8, Spec 87.5, AUC 0.90 (CI 0.82–0.98) |
| Jiang, 2014 ^28^ | PC | China  [In Chinese] | 2010-13 | TH | ≥36 wk;  Fetal brady >5min/ intrapartum event;  Apgar ≤3 at 1min and ≤5 at 5min/ cord art pH ≤7.0  + Neurology abnormal | 31  NDI: 6 | 15-18m: NDI- BSID (PDI/MDI <80) | GFAP >0.07ng/ml, 6-12h | NDI vs no NDI:  Sens 77, Spec 78, AUC 0.71 (CI 0.70-0.77) |
| Velazquez, 2014 ^29^ | PC | Cuba  [In Spanish] | 2011-12 | None | Apgar ≤3 at 1 min + <7.0 at 5min;  pH <7.3;  + Neurology abnormal; signs of organ involvement | 25  NDI: 12 | 12m: NDI- Gesell Scale (mod-severe) | Arterial pH <1hr:  <7.00  7.00 - 7.14  7.15 - 7.29 | NDI vs no NDI- n:  8/12 vs 0/13  4/12 vs 6/13  0/12 vs 7/13 |
| Liu, 2010 ^30^ | P | China | 2005-08 | None | Term;  Apgar 3 at 1 min and <5 at 5 min / cord art pH≤7.0/ postnatal pH<7.20 and BE<10.0;  + Neurology abnormal (seizures, coma, hypotonia, irritation, reflexes. pupils); multisystem organ dysfunction | 44  14 | 12m: NDI- DDST II, CP | Cord blood IL-1b | Sens 89, Spec 81, p<0.01 |
| Khedr, 2009 ^13^ | PCC | Egypt | ? | None | >37 wk;  Abnormal FHR / mec;  Apgar 5min <7/ 1st art pH <7.20/ delayed resp >5min/ resus at birth;  + Neurology abnormal (Thompson) | 20  Death: 1  NDI: 5 | 12m: Death, or NDI- CP, severe motor dysfunction | Arterial pH, cord:  <7.1  ≤7.0 | Death/NDI vs favourable outcome, n:  6/6 vs 2/14 - TP=6, FP=2, FN=0, TN=12  4/6 vs 2/14 - TP=4, FP=2, FN=2, TN=12 |
| **Author, year** | **Study design** | **Country** | **Recruitment years** | **Neuro-protection (n)** | **NE definition** | **Total no*;**  **No. with death/NDI** | **Outcome: duration of follow-up, definition** | **Predictor** | **Statistical association with outcome** |
| ***e. Postneonatal neurological assessments*** | | | | | | | | | |
| Tran, 2024 ^2^ | P | Vietnam | 2016-19 | TH | ≥36 wk, <6h old;  Apgar ≤5 or resus at 10 min  + Neurology abnormal (Sarnat);  + aEEG abnormal background or seizures | 69  CP: 19 | 18m: CP (Bax criteria) | HINE, 6m:  <40 | Sens 68, Spec 98, PPV 93, NPV 89, AUC 0.90 (95% CI 0.81-0.96) |
| Zhussupova, 2024 ^31^ | PC | Kazakhstan | 2020-21 | TH (14 ) vs none | Term;  Apgar <5 at 5min and <6 at 10min/ resus >10min/ pH ≤7.0/ BE >-16/ lac >10;  + Neurology abnormal (Sarnat) | 31  CP: 9 | 24m: CP | GMA:  3-4 wk- Cramped  synchronised  12-14 wk- Absent fidgety  HINE, 3m- score <67 | CP vs no CP, n:  3/9 vs 0/22 - TP=3, FP=0, FN=6, TN=22  8/9 vs 0/22 - TP=8, FP=0, FN=1, TN=22  8/9 vs 1/22 - TP=8, FP=0, FN=1, TN=22 |
| Aker, 2022 ^21^ | RCT | India | 2013-15 | TH (50%) vs none | ≥35 wk, BW >1.8kg, <6h old;  Apgar ≤5 or resus at 10 min/ cord or 1^st^ hr pH <7.00 or BE ≥-12;  + Neurology abnormal (NICHD) | 38  Death: 1  NDI: 8 | 18m: Death, or NDI- BSID-III (cognitive/ motor <85), CP (GMFCS 3-5), vision/ hearing loss, seizures | GMA, 10-15 wk:  Absent fidgety movements  Sporadic or absent fidgety  movements | Death/NDI vs favourable outcome:  6/9 vs. 4/29  Sens 60.0 (CI 26.2-87.8), Spec 89.3 (71.8-97.7), PPV 66.7 (38.0-86.7), NPV 86.2 (74.3-93.1), accuracy 81.6 (65.7-92.3)  Death/NDI vs favourable outcome:  7/9 vs. 13/29  Sens 70.0 (34.8-93.3), Spec 57.1 (37.2-75.5), PPV 36.8 (24.4-51.3), NPV 84.2 (66.3 to 93.6), accuracy 60.5 (43.4-76.0) |
| Apaydin, 2021 ^22^ | RC | Turkey | 2014-18 | TH (34) vs. head cooling ( 13) | ≥37 wk;  Apgar <5 at 5 min/ abnormal FHR/ mec/ pH *<*7.0  + Neurology abnormal (Sarnat) | 47  NDI: 9  (CP:6) | 24m: NDI- BSID-II (MDI/PDI <85), CP | GMA, 12-16 wk:  Absent fidgety movements  HINE, 3-6m:  <67 | For CP:  Sens 83.3, Spec 100, AUC 0.92  For NDI (BSID-II PDI):  Sens 92.9, Spec 100, AUC 0.99  For NDI (BSID-II MDI):  Sens 90.5, Spec 80, AUC 0.84  For CP:  Sens 83.3, Spec 100, AUC 0.92  For NDI (BSID-II PDI):  Sens 92.9, Spec 100, AUC 0.99  For NDI (BSID-II MDI):  Sens 90.5, Spec 80, AUC 0.84  NDI (BSID-II PDI/MDI) vs no NDI, n:  7/8 vs 5/39 – TP=7, FP=5, FN=1, TN=34 |
| Soleimani, 2015 ^32^ | PC | Iran | 2012-13 | None | ≥35 wk;  Resus at birth/ Apgar <5 at 5 min/ mec/ abnormal FHR/ pH <7.0 or BE≥−16;  + Neurology abnormal (Sarnat) | 15  NDI: 6 | 12-18m: NDI- Infanib (>-2SD below mean; mod-severe) | GMA, 3m:  Absent fidgety movements | NDI vs no NDI- n:  5/6 vs 3/9 - Sens 83 (CI 35.9–99.6), Spec 66 (CI 29.9–92.5) |

*Neuroprotection (n)= for studies implementing TH in a proportion of the cohort, the number or % of infants are displayed where data were provided. **Total N= total number of infants with complete data for both predictor and outcome reported.

Sensitivity, specificity, PPV, and NPV values are in %. Data were rounded to 1 decimal place, where ≥2 decimal places were reported.

*Abbreviations: ADSI= Ankara Developmental Screening Inventory; aEEG=amplitude-integrated electroencephalography;* *ASQ=Ages & Stages Questionnaire; art=arterial; BE=base excess; BG=background; brady=bradycardia; BSID II/ III=Bayley Scales of Infant and Toddler Development 2^nd^/3^rd^ edition, CDCC= Children’s Development Center of China; CI=95% confidence interval; CP=cerebral palsy, CTG=cardiotocograph; DAS-II=Developmental Assessment Scale for Indian Infants- 2^nd^ edition; DDST-II=Denver Developmental Screening Test 2^nd^ edition; DQ=developmental quotient; DTI=diffuse tensor imaging; DWI=diffusion-weighted imaging; EEG=electroencephalography; Epo=Erythropoietin; FA=fractional anisotropy; FHR=fetal heart rate; Gesell DS=Gesell Developmental Schedules; GFAP=glial fibrillary acidic protein; GMA=Prechtl’s General Movements Assessment; GMDS-II=Griffiths Mental Development Scales 2^nd^ edition; GMFCS= Gross Motor Function Classification System; HINE=Hammersmith Infant Neurological Examination; hr=hour; Infanib=Infant Neurological International Battery test; IL=interleukin; kg=kilogram; lac=lactate; mec=meconium; MDI: Mental Developmental Index; min=minute; mod=moderate; m=month; MRI=magnetic resonance imaging; MRS=magnetic resonance spectroscopy; NDI=neurodevelopmental impairment; NE=neonatal encephalopathy; nRBC=nucleated red blood cells; OFC=occipito-frontal circumference; PC=prospective cohort; PCC=prospective case-control; PDI: Psychomotor Developmental Index; RC=retrospective cohort; RCT=randomised controlled trial; resp=respiration; resus=resuscitation; Sarnat=Modified Sarnat staging; SD=standard deviation; sens= sensitivity; spec= specificity; TH=therapeutic hypothermia; US= ultrasound; wk=week; yr=year*

References :

1. Mathieson SR, Nanyunja C, Sadoo S, Nakalembe S, Duckworth E, Muryasingura S, et al. EEG background activity, seizure burden and early childhood outcomes in neonatal encephalopathy in Uganda: a prospective feasibility cohort study. EClinicalMedicine. 2024;78:102937.

2. Tran HTT, Le HT, Tran DM, Nguyen GTH, Hellstrom-Westas L, Alfven T, et al. Therapeutic hypothermia after perinatal asphyxia in Vietnam: medium-term outcomes at 18 months - a prospective cohort study. BMJ Paediatrics Open. 2024;8(1):21.

3. Huang HZ, Hu XF, Wen XH, Yang LQ. Serum neuron-specific enolase, magnetic resonance imaging, and electrophysiology for predicting neurodevelopmental outcomes of neonates with hypoxic-ischemic encephalopathy: a prospective study. BMC Pediatr. 2022;22(1):290.

4. Boskabadi H, Maamouri G, Zakerihamidi M, Bagheri F, Mashkani B, Mafinejad S, et al. Interleukin-6 as A Prognostic Biomarker in Perinatal Asphyxia. Iranian Journal of Child Neurology. 2021;15(3):119-30.

5. Montaldo P, Cunnington A, Oliveira V, Swamy R, Bandya P, Pant S, et al. Transcriptomic profile of adverse neurodevelopmental outcomes after neonatal encephalopathy. Scientific Reports. 2020;10(1):13100.

6. Preeti S, Kadam A, Kadam S, Vaidya U, Kumar P, Bhagat I, et al. Anthropometric measures as biomarkers of neurodevelopmental outcomes of newborns with moderate to severe hypoxic ischemic encephalopathy. Journal of Neonatal-Perinatal Medicine. 2019;12(2):127-34.

7. Tann CJ, Webb EL, Lassman R, Ssekyewa J, Sewegaba M, Musoke M, et al. Early Childhood Outcomes After Neonatal Encephalopathy in Uganda: A Cohort Study. EClinicalMedicine. 2018;6:26-35.

8. Malla RR, Asimi R, Teli MA, Shaheen F, Bhat MA. Erythropoietin monotherapy in perinatal asphyxia with moderate to severe encephalopathy: a randomized placebo-controlled trial. Journal of Perinatology. 2017;37(5):596-601.

9. Kali GT, Martinez-Biarge M, Van Zyl J, Smith J, Rutherford M. Therapeutic hypothermia for neonatal hypoxic-ischaemic encephalopathy had favourable outcomes at a referral hospital in a middle-income country. Acta Paediatrica. 2016;105(7):806-15.

10. Lally PJ, Price DL, Pauliah SS, Bainbridge A, Kurien J, Sivasamy N, et al. Neonatal encephalopathic cerebral injury in South India assessed by perinatal magnetic resonance biomarkers and early childhood neurodevelopmental outcome. PLoS ONE [Electronic Resource]. 2014;9(2):e87874.

11. Polat M, Simşek A, Tansuğ N, Sezer RG, Ozkol M, Başpınar P, et al. Prediction of neurodevelopmental outcome in term neonates with hypoxic-ischemic encephalopathy. Eur J Paediatr Neurol. 2013;17(3):288-93.

12. Ong LC, Kanaheswari Y, Chandran V, Rohana J, Yong SC, Boo NY. The usefulness of early ultrasonography, electroencephalography and clinical parameters in predicting adverse outcomes in asphyxiated term infants. Singapore Med J. 2009;50(7):705-9.

13. Khedr E, Hamed S, Mohammed GaSa, El Attar A. A preliminary study of multimodal evoked potentials in relation to outcome in term infants with post-asphyxial hypoxic-ischemic encephalopathy. Journal of Pediatric Neurology. 2009;07:265 - 73.

14. El-Ayouty M, Abdel-Hady H, El-Mogy S, Zaghlol H, El-Beltagy M, Aly H. Relationship between electroencephalography and magnetic resonance imaging findings after hypoxic-ischemic encephalopathy at term. Am J Perinatol. 2007;24(8):467-73.

15. Belet N, Belet U, Incesu L, Uysal S, Ozinal S, Keskin T, et al. Hypoxic-ischemic encephalopathy: correlation of serial MRI and outcome. Pediatr Neurol. 2004;31(4):267-74.

16. Xiqun J, Cuiqing LIU, Yaofang XIA, Li MA. The early diagnostic and prognostic value of amplitude integrated electroencephalography in neonates with hy-poxic-ischemic encephalopathy. Chinese Journal of Applied Clinical Pediatrics. 2015(24):1102-5.

17. Cseko AJ, Bango M, Lakatos P, Kardasi J, Pusztai L, Szabo M. Accuracy of amplitude-integrated electroencephalography in the prediction of neurodevelopmental outcome in asphyxiated infants receiving hypothermia treatment. Acta Paediatrica, International Journal of Paediatrics. 2013;102:707-11.

18. Jose A, Matthai J, Paul S. Correlation of EEG, CT, and MRI Brain with Neurological Outcome at 12 Months in Term Newborns with Hypoxic Ischemic Encephalopathy. J Clin Neonatol. 2013;2(3):125-30.

19. Liu DL, Shao XM, Wang JM. [Amplitude-integrated electroencephalographic monitoring in early diagnosis and neurological outcome prediction of term infants with hypoxic-ischemic encephalopathy]. Zhonghua Er Ke Za Zhi. 2007;45(1):20-3.

20. Al-Garni A, Aslam S, Assis Z, Avanaki K, Bagnato MC, Bainbridge A, et al. Proceedings of the 15th International Newborn Brain Conference: Neuro-imaging studies: Fota Island, Cork, Ireland, February 28th – March 2nd 2024. Journal of Neonatal-Perinatal Medicine. 2024;17(3):S421-S45.

21. Aker K, Thomas N, Adde L, Koshy B, Martinez-Biarge M, Nakken I, et al. Prediction of outcome from MRI and general movements assessment after hypoxic-ischaemic encephalopathy in low-income and middle-income countries: data from a randomised controlled trial. Archives of Disease in Childhood Fetal & Neonatal Edition. 2022;107(1):32-8.

22. Apaydın U, Erol E, Yıldız A, Yıldız R, Acar Ş S, Gücüyener K, et al. The use of neuroimaging, Prechtl's general movement assessment and the Hammersmith infant neurological examination in determining the prognosis in 2-year-old infants with hypoxic ischemic encephalopathy who were treated with hypothermia. Early Hum Dev. 2021;163:105487.

23. el beheiry A, Elgamal M, Ettaby A, Omar T, Badeib A. Can diffusion tensor imaging predict cerebral palsy in term neonates with hypoxic ischemic encephalopathy? Egyptian Journal of Radiology and Nuclear Medicine. 2019;50.

24. Kalay S, Öztekin O, Tezel G, Çetiner İ, Turan S, Akçakuş M, et al. Comparison of the efficacy of diffusion-weighted magnetic resonance imaging and conventional magnetic resonance imaging in determining the prognosis in newborns with hypoxic ischemic encephalopathy. Turk Arch Pediatr 2011;46: 292-295.

25. Mfingwana L, van Zyl J, Smith J, Rutherford M, Kali GTJ. Nucleated red blood cells in neonates with hypoxic ischaemic encephalopathy treated with hypothermia: A worthwhile prognostic biomarker for clinicians in LMIC? SAJCH South African Journal of Child Health. 2023;17(3):122-6.

26. Pang R, Mujuni BM, Martinello KA, Webb EL, Nalwoga A, Ssekyewa J, et al. Elevated serum IL-10 is associated with severity of neonatal encephalopathy and adverse early childhood outcomes. Pediatric Research. 2022;92(1):180-9.

27. Gane BD, Bhat V, Rao R, Nandhakumar S, Harichandrakumar KT, Adhisivam B. Effect of therapeutic hypothermia on DNA damage and neurodevelopmental outcome among term neonates with perinatal asphyxia: a randomized controlled trial. Journal of tropical pediatrics. 2014;60(2):134‐40.

28. Shu-Hong J, Jin-Xiu W, Yi-Ming Z, Hui-Fen J. Effect of hypothermia therapy on serum GFAP and UCH-L1 levels in neonates with hypoxic-ischemic encephalopathy. Chinese Journal of Contemporary Pediatrics. 2014(12):1193-6.

29. Velázquez NH, Borjas IL, Matos AA, editors. Evaluación neurológica en recién nacidos con asfixia al nacer2014.

30. Liu J, Feng ZC. Increased umbilical cord plasma interleukin-1 beta levels was correlated with adverse outcomes of neonatal hypoxic-ischemic encephalopathy. J Trop Pediatr. 2010;56(3):178-82.

31. Zhussupova Z, Jaxybayeva A, Ayaganov D, Tekebayeva L, Mamedbayli A, Tamadon A, et al. General movement assessment efficacy for assessment of nervous system integrity in children after hypoxic-ischemic encephalopathy in middle income countries. Early Hum Dev. 2024;192:105992.

32. Soleimani F BRSMABAMA. General movements as a predictive tool of the neurological outcome in term born infants with hypoxic ischemic encephalopathy.

Supplementary material 5: Meta-analyses of prevalence of adverse early childhood outcome after NE in included articles: [A] Composite death and neurodevelopmental impairment [B] Neurodevelopmental impairment (in survivors)

[A] Death and neurodevelopmental impairment

[B] Neurodevelopmental impairment in survivors

Where studies presented prevalence of both death/NDI and NDI outcomes, only death/NDI was included. Where studies reported more than one predictor, with different sample sizes, the largest sample size was included. Two studies [Pang 2022, Nanyunja 2024] were omitted from the prevalence estimates, as their cohorts overlapped with other included studies [Tann 2018 and Mathieson 2024, respectively]. Total=total number of infants with complete data for predictor and outcome, providing the denominator for prevalence calculation. *Abbreviations: CI=confidence interval; ES=effect size (ie. prevalence); NDI=neurodevelopmental impairment;* *TH= therapeutic hypothermia.*

Supplementary material 6: Risk of bias assessment for included articles

| **Author, year** | **Participants** | **Study attrition** | **Prognostic factor measurement** | **Outcome measurement** | **Statistical analysis and presentation** | **Overall RoB** |
| --- | --- | --- | --- | --- | --- | --- |
| **Aker, 2022** ^21^ |  |  |  |  |  |  |
| **Apaydin, 2021** ^22^ |  |  |  |  |  |  |
| **Belet, 2004** ^15^ |  |  |  |  |  |  |
| **Boskabadi, 2021** ^4^ |  |  |  |  |  |  |
| **Cseko, 2013** ^17^ |  |  |  |  |  |  |
| **El Ayouty, 2007** ^14^ |  |  |  |  |  |  |
| **El Beheiry, 2019** ^23^ |  |  |  |  |  |  |
| **Gane, 2014** ^27^ |  |  |  |  |  |  |
| **Huang, 2022** ^3^ |  |  |  |  |  |  |
| **Jia, 2014** ^16^ |  |  |  |  |  |  |
| **Jiang, 2014** ^28^ |  |  |  |  |  |  |
| **Jose, 2013** ^18^ |  |  |  |  |  |  |
| **Kalay, 2011** ^24^ |  |  |  |  |  |  |
| **Kali, 2016** ^9^ |  |  |  |  |  |  |
| **Khedr, 2009** ^13^ |  |  |  |  |  |  |
| **Lally, 2014** ^10^ |  |  |  |  |  |  |
| **Liu, 2007** ^19^ |  |  |  |  |  |  |
| **Liu, 2010** ^30^ |  |  |  |  |  |  |
| **Malla, 2017** ^8^ |  |  |  |  |  |  |
| **Mathieson, 2024** ^1^ |  |  |  |  |  |  |
| **Mfingwana, 2023 ^21^** |  |  |  |  |  |  |
| **Montaldo 2020** ^5^ |  |  |  |  |  |  |
| **Nanyunja, 2024** ^20^ |  |  |  |  |  |  |
| **Ong, 2009**^12^ |  | **.** |  |  |  |  |
| **Pang, 2021** ^26^ |  | **.** |  |  |  |  |
| **Polat, 2013** ^11^ |  |  |  |  |  |  |
| **Preeti, 2019** ^6^ |  |  |  |  |  |  |
| **Soleimani, 2015** ^32^ |  |  |  |  |  |  |
| **Tann, 2018** ^7^ |  |  |  |  |  |  |
| **Tran, 2024** ^2^ |  |  |  |  |  |  |
| **Velazquez, 2014** ^29^ |  |  |  |  |  |  |
| **Zhussupova, 2024** ^31^ |  | **.** |  |  |  |  |

*Reference: Hayden JA, van der Windt DA, Cartwright JL, Cote P, Bombardier C. Assessing bias in studies of prognostic factors. Ann Intern Med. 2013 Feb 19;158(4):280–6.*

*Abbrevations: RoB=risk of bias.* Green boxes=low RoB; orange boxes=moderate RoB; red boxes=high RoB. For overall RoB rating, low RoB was defined as all domains low RoB/ up to one moderate RoB; high was defined as one or more domains high/ ≥3 moderate RoB; moderate RoB was assigned for all articles in between.

See reference list in supplementary material 4 for full bibliography of individual studies.
